# Eureka-DMA: an easy-to-operate graphical user interface for fast comprehensive investigation and analysis of DNA microarray data

**DOI:** 10.1186/1471-2105-15-53

**Published:** 2014-02-24

**Authors:** Sagi Abelson

**Affiliations:** 1 The Ruth and Bruce Rappaport Faculty of Medicine, Haifa, Technion, Israel

**Keywords:** GUI, Software, Microarray, Analysis, Differential expression, Pathways, Gene ontology

## Abstract

**Background:**

In the past decade, the field of molecular biology has become increasingly quantitative; rapid development of new technologies enables researchers to investigate and address fundamental issues quickly and in an efficient manner which were once impossible. Among these technologies, DNA microarray provides methodology for many applications such as gene discovery**,** diseases diagnosis, drug development and toxicological research and it has been used increasingly since it first emerged. Multiple tools have been developed to interpret the high-throughput data produced by microarrays. However, many times, less consideration has been given to the fact that an extensive and effective interpretation requires close interplay between the bioinformaticians who analyze the data and the biologists who generate it. To bridge this gap and to simplify the usability of such tools we developed Eureka-DMA — an easy-to-operate graphical user interface that allows bioinformaticians and bench-biologists alike to initiate analyses as well as to investigate the data produced by DNA microarrays.

**Results:**

In this paper, we describe Eureka-DMA, a user-friendly software that comprises a set of methods for the interpretation of gene expression arrays. Eureka-DMA includes methods for the identification of genes with differential expression between conditions; it searches for enriched pathways and gene ontology terms and combines them with other relevant features. It thus enables the full understanding of the data for following testing as well as generating new hypotheses. Here we show two analyses, demonstrating examples of how Eureka-DMA can be used and its capability to produce relevant and reliable results.

**Conclusions:**

We have integrated several elementary expression analysis tools to provide a unified interface for their implementation. Eureka-DMA's simple graphical user interface provides effective and efficient framework in which the investigator has the full set of tools for the visualization and interpretation of the data with the option of exporting the analysis results for later use in other platforms. Eureka-DMA is freely available for academic users and can be downloaded at http://blue-meduza.org/Eureka-DMA.

## Background

Since it was first introduced, the use of microarray technology has been growing rapidly. The capacity to survey the expression of thousands of genes in a single experiment was proven to be extremely valuable for gene expression profiling in many fields including basic biological studies; medical diagnostics and personalized medicine; drug discovery and development; toxicogenomics; and cancer research [1-3]. Gene expression analysis has moved well beyond the simple goal of identifying few genes of interest. Other algorithms - for data visualization and profiling, for assessing the involvement of genes in particular molecular pathways and for searching for the enrichment of a common ontology - have all become important approaches, both for the pursuit of hypothesis-driven inquiries and for the generation of new hypotheses. Therefore, efficient analysis and interpretation of the high volume data that is produced from microarrays represents a major challenge; it may become the most formidable obstacle, which biologists face once trying to extract meaningful information from their experiments. Many software-products specifically designed for microarray analysis are available. These range from free simple programs that preform several basic tasks with relatively limited scientific benefit to comprehensive programs, which usually require prior knowledge in statistics; these are also usually expensive and their complicated usability usually restricts their usage to researchers already familiar with the program's interface. Eureka-DMA is an application that combines the simplicity of operation and data management with the executions of multiple analysis tasks to transform the high throughput data into meaningful and understandable information. Eureka-DMA provides algorithms for searching genes with differential expression between groups, searching for enrichment of pathways from KEGG PATHWAY database (*http://www.genome.jp/kegg/pathway.html*) and enrichment of Gene Ontology (GO) terms from the Gene Ontology database (*http://www.geneontology.org*). Furthermore, it incorporates options for the analysis of time series datasets and expression profiling; all implemented, in an explicit, easy-to-use graphical user interface (GUI), designed to provide intuitive control throughout data processing. The accessibility and simplicity of Eureka-DMA, combined with the set of bioinformatics tools for full comprehensive analysis should address a wide range of scientists: specialists, as well as enthusiastic researchers with no prior knowledge in bioinformatics.

## Implementation

### General software descriptions, aspects and design

Eureka-DMA software was written in MATLAB programming language and can be run as a GUI in the MATLAB environment or as a standalone Microsoft Windows executable. Currently, multiple effective microarray analysis algorithms are available as MATLAB functions but their usability is restricted to researchers experienced in computer science and familiar with MATLAB's computing environment. In order to provide a unified platform for an extensive interpretation of microarray data, we integrated several MATLAB-analysis tools - either as is or with slight modifications to allow proper usage with Eureka-DMA's GUI - with new tools. Eureka-DMA's main GUI enables simple and quick control throughout the elucidation processes of the input data by the interaction with several other sub-GUIs, all designed to operate with limited user-input. Eureka-DMA's main GUI also includes “ToolTip” explanation elements for the different options, helping less advanced users understand the purposes of the different software components. For programmers and bioinformaticians who might wish to edit the code, the script contains detailed explanations for the embedded functions and is designed to enable easy understanding along with easy accessibility for the implementation of modifications. Last but not least, Eureka-DMA provides with functional annotation files for mouse (*M.musculus*) and human, allowing the integration of a standalone gene description tool, which enables the withdrawal of the gene's summary paragraph from the NCBI gene database (*http://www.ncbi.nlm.nih.gov/gene*) into the main GUI and to generate description reports about genes of interest. The appropriate species is recognized automatically according to the user data file and does not require user input.

### Loading and exporting of data

It is necessary that raw data will be input as Windows Excel or text files. These familiar file formats spare users from dealing with multiple and less common microarray files received by different manufacturers and can also be easily obtained by exportation from the various microarray manufacturer company's softwares. When pulling data from microarray databases, such as Gene Expression Omnibus (GEO) [4] and ArrayExpress [5], small manual adjustments may be required. The download package provides example data files that can be used either as a template or to experiment with Eureka-DMA different options. Results can be exported at any time during the analysis into ‘xls’, Windows Excel or ‘txt’ file formats for further process of the data with other external tools. Furthermore, the different visualization tools enable exportation into common figure formats.

### Normalization of Raw data

Microarrays are intended to detect the expression level variation between different samples, making normalization an important preceding step to any analysis. A number of normalization methods have been designed to address the many reasons which can lead to non-biological inconsistency between samples. Quantile normalization is one method which is frequently used when interpreting microarray data [6]. Subject to user decision, when a set of arrays is available, this method will make the distribution of the intensities for each array the same; this process can be tracked with the visualization box plot tool in Eureka-DMA main GUI.

### Filtering Non-relevant data

Eureka-DMA offers two options for subtraction of transcripts which are expressed below background intensities. 1) User defined p-value threshold: Raw data is comprised with manufacturer specific probe IDs assigned to internal controls for the calculation of detection p-values [7]. When detection p-values are available, the user can set a threshold to filter transcripts with high detection p-values. 2) User defined intensity threshold: In case that detection p-values are not available, the user can estimate and set an intensity threshold to filter transcripts with low intensities, based on prior knowledge (e.g., genes expressed only in brain tissue will have low intensities when liver tissue is tested) or observation of the raw data for the internal negative controls [8]. It is to be noted that for both options, subtraction of transcripts is done only when all the samples do not meet the criteria, thus avoiding the elimination of those transcripts which did not get detected in only part of the samples.

### Detecting differential expression among conditions

Eureka-DMA provides several options for the detection of genes that exhibit sufficient variation across measured conditions. Volcano plot-display of significance versus gene expression ratio (fold change) can be created in cases where the data can be divided into two groups*,* i.e. experimental group and control group [9]*.* Updating the main GUI for those genes that are considered to be significantly differentially expressed can be initiated through the volcano plot sub-GUI by the user adjustment of the fold change and/or p-value cutoffs. When the data contain more than two conditions, e.g. time series data, a differential expression can be defined by measuring the correlation coefficient r as

$$r=\frac{\sum_{m}\sum_{n}\left(A_{\mathrm{mn}}-\bar{A}\right)\left(B_{\mathrm{mn}}-\bar{B}\right)}{\sqrt{\left(\sum_{m}\sum_{n}{\left(A_{\mathrm{mn}}-\bar{A}\right)}^{2}\right)\left(\sum_{m}\sum_{n}{\left(B_{\mathrm{mn}}-\bar{B}\right)}^{2}\right)}}$$

Where A = vector of 1 through the number of samples and B = vector of the gene's intensities across samples. Genes that pass the user defined r threshold will be displayed by a rank order, determined by the intra-group variation and the differential expression of the gene between the groups. Additionally, Eureka-DMA provides a filtering option based on minimal variation criteria across samples [8].

### Pathway enrichment analysis

Mapping genes on cellular pathways is one of the primary goals in the analysis of transcriptomic data and is very important for inferring biological mechanisms of diverse biological conditions. Eureka-DMA contains a statistical feature that seeks biological and chemical pathways from KEGG pathway database that are significantly over-represented in the user's gene list. With respect to a background set of genes, hypergeometric probability density test [10] is conducted to check whether the number of differentially expressed genes from the user's data was greater than expected by chance. Nominal p-values are assigned and the user can browse through the enriched pathways and create an illustration of the pathway with the differentially expressed genes highlighted. It is to be noted that this analysis can be initiated with any list of genes and is not dependent on any previous analyses.

### Functional enrichment of gene ontology

After identifying the differentially expressed genes, one will wish to ascribe them some biological meaning. Identification of significant co-clustered genes with similar properties (i.e. shares a cellular component, a biological process, or a molecular function) can be achieved with the ‘Gene Ontology’ gene annotation scheme. Eureka-DMA will apply the hypergeometric probability density test [10] to pinpoint GO categories that are statistically over-represented in the set of genes defined by the user's previous analyses. Results may be illustrated as hierarchical graphical displays, which provide the summarization of the significance GO terms. Updating the GO database version can be done automatically by user's choice.

### Clustering, classification and visualization tools

Clustering and classification algorithms applied to high dimensional expression data has the potential to provide deeper insight to the underlying data structure and is commonly used when *a priori* knowledge about specific subgroups is lacking. Eureka-DMA supports several widely used clustering and classification algorithms such as hierarchical and K-means clustering [11-13], as well as probabilistic principal component analysis (PPCA) [14], all of which can be used in case the data suffer from missing values [15]. Eureka-DMA also provides the following visualization tools: an interactive volcano plot that demonstrates genes with differential expression; a heat-map accompanying the hierarchical clustering analysis; a gene ontology bio-graph showing the terms and their ancestry; an illustration of enriched KEGG pathways; a bar plot; and, finally, box plots that display the differences between all the analyzed entries across all samples or the differences between single entries across the user defined groups. The variety of the provided visualization tools can be utilized separately for their intended use or in combination for quality control and analysis assessment.

### Primer design for RT-qPCR validation

Reverse transcription quantitative PCR (RT-qPCR) is often used to validate gene expression measurements from DNA microarray experiments. Eureka-DMA offers a primer design tool that can either import the list of genes from the software's main GUI or used as a standalone without the requirement of any previous analyses. The primer design GUI contains various execution options: 1) cDNA sequence data can be imported directly from GenBank (*http://www.ncbi.nlm.nih.gov/genbank)* or be entered manually. 2) The user can choose to apply adjustable filters that control the desire primer's length, G and C nucleotide content, and melting temperature. 3) The user can choose to use additional parameters to filter primers that contain nucleotide repeats and primers that might form secondary structures such as hairpins, dimers and cross dimerization. 4) The length of the desire transcripts is controllable. The impact of individual filters can be observed in a 2 dimensional plot that can also reveal the existence of problematic regions within the cDNA transcript.

## Results and discussion

Eureka-DMA has been tested on a number of data sets in order to assess the program’s capability to deliver meaningful and relevant biological insights regarding the analyzed data. The results of two such analyses demonstrating the feasibility of Eureka-DMA are reported here. The first dataset contains expression data of 83 colorectal cancer (CRC) patients, divided into two groups: responders and non-responders to FOLFOX chemotherapy. The second dataset is obtained from a time series study which assesses the global changes in gene expression patterns, in the lungs of mice infected with influenza virus, over a period of 60 days.

### Analysis of colorectal tumors of responders and Non-responders to FOLFOX chemotherapy

**Figure 1:**
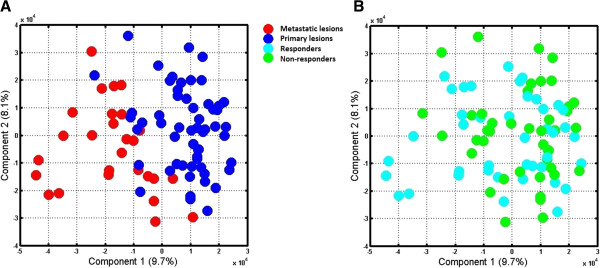
**Data summarization by probabilistic principal component analysis.** PPCA results are provided as two-dimensional representations based on the contribution scores of the first two components. **(A)** Discrimination between metastatic lesions and primary tumors and **(B)** between responders and non-responders to FOLFOX therapy is shown as indicated in the color capture.

Publicly available raw expression data for 83 CRC generated using the Affymetrix Human Genome U133 Plus 2.0 platform was downloaded from the GEO online database [4] as two separate DataSet SOFT files. This study [16] describes tumor samples - including 56 primary CRCs and 27 metastatic lesions - that were obtained from different patients before therapy. The investigators divided the samples into training and test sets. The training set was used to identify classifier genes that are able to predict responders to FOLFOX therapy; after which the classifier signature was used on the test set in order to evaluate its sensitivity and specificity. Random Forests algorithm [17] was used for the identification of the predictor genes. Since the classifier group of genes was supported by limited biological meaning, we independently applied Eureka-DMA and conducted detailed biological analysis and interpretation. The merged training and test sets data was loaded into Eureka-DMA and subjected to normalization. Based on the intensity values of the chip’s internal negative controls, probes with intensity values below 90 across all samples were filtered out. Expression profiles of the 31826 remaining probes, compared across samples by PPCA, revealed obvious separation between primary tumors and metastatic lesions (Figure 1A), placing them in separable regions in the reduced dimensional space. However, when highlighting samples from response and non-response patients (Figure 1B) we observed no distinguishable clusters. Additionally, there was no indication of apparent relation in responsiveness to FOLFOX with primary nor metastatic lesions (Figure 1A and B). By using Eureka-DMA's volcano plot tool, we identified a list of 77 probes that are differentially expressed between responders and non-responders samples by at least 2 fold, at significant levels of p-value < 0.05 (Additional file 1, Figure 2). Interestingly, the 14 classifier genes publish in the original study were effectively able as a set to stratify responders to FOLFOX therapy with high accuracy [16]; however, when checked individually one could see that the expression of these genes only slightly differed between the responders and non-responders groups (fold change < 1.5, p-value < 0.05). Therefore, we are providing here a completely new elucidation of the data and we are strengthening our interpretation with robust published evidence. By investigating the differentially expressed genes with Eureka-DMA's gene description tool we notice that the list can be divided into 3 major groups, all of which contain transcripts with a higher expression level in the non-responders category (Additional file 1, Figure 2). Group 1 consists of 5 probes corresponding to 4 genes. *CYP2C9*, *CYP2E1* and *CYP2C8* belong to cytochrome P450s superfamily of proteins, which represents a large class of heme-containing enzymes that catalyzes the metabolism of multitudes of exogenous substrates and participates in their inactivation [18]. *FMO3* belong to the flavin-containing monooxygenase enzyme family involved in the oxidation of nucleophilic atoms, which produce more polar substances as a prelude to excretion [19]. Group 2 consist of 40 probes corresponding to 31 genes that encode different serum proteins. In a recently published paper by Hyung et al. [20], profiling of N-glycosylated proteins in the serum of advanced breast cancer patients was performed in order to discover serum biomarkers for chemoresistance. Twenty three proteins were identified to be differentially expressed between patients defined as sensitive and patients defined as resistant to docetaxel and doxorubicin treatment. The expression pattern of several proteins was later validated in independent samples. Interestingly, in our analysis we found transcripts encodes to 10 out of the 23 aforementioned proteins (Additional file 1). Even though patients bearing different type of cancer and under different treatment regimen were investigated, it is very unlikely that these results are consequence of chance (p = 9.29e-20 by hypergeometric probability density test); This is further supported by other studies, which demonstrate elevated expressions of different serum proteins found in our analysis and in patients with chemoresistant tumors [21,22]. Moreover, since different profiling methods were used in the two studies - peptide separation and quantification from patients serum using LC/MS/MS compared with GeneChip microarray of tumor samples - our results also imply that the high levels of several serum proteins found at Hyung et al. study in chemoresistant patient's blood (Figure 3), might be due to the consequence of biological processes that occurred in the tumors rather than patient*-*inherent causes. Group 3 consists of 12 probes, corresponding to 10 genes, all related to lipid metabolism, transport and/or synthesis. Even though two genes in this group - *APOA1* and *AMBP* were also identified to be elevated in paclitaxel resistance ovarian cell lines [23], the relevance and contribution of these genes to the resistance phenotype as a group is yet to be determined. By using Eureka-DMA pathway enrichment analysis tool we were able to further support our unbiased categorization of the genes into the 3 groups. By setting a threshold of p-value < 0.05 we found enrichment of 17 pathways (Additional file 2) including the complement and coagulation cascades (Figure 4) and PPAR signaling pathway, which were described before to be dysregulated in several tumor types [24] and have been implicated with the formation of resistant prostate cancer in mice [25]. Moreover we found enrichment of pathways involved in xenobiotic and drug metabolism along with multiple other metabolic pathways. This implies that drug resistance in CRC may partially be acquired by processes that regulate drug activity and availability. Taken together, the analysis of the data using Eureka-DMA exposes reasonable and relevant information regarding the comparison made here. Literature mining of several differential genes and pathways, which were highlighted by our analysis, revealed their aberrant regulation in other cancer types under other treatment regimens, suggesting common mechanisms that may regulate drug resistance. Given the strong statistical enrichment of the complement and coagulation pathways along with elevated expression of several drug metabolism genes, suggest the importance of a further examination of the exact underlying molecular and metabolic processes as they may hold the key for understanding FOLFOX resistance in CRC.

**Figure 2:**
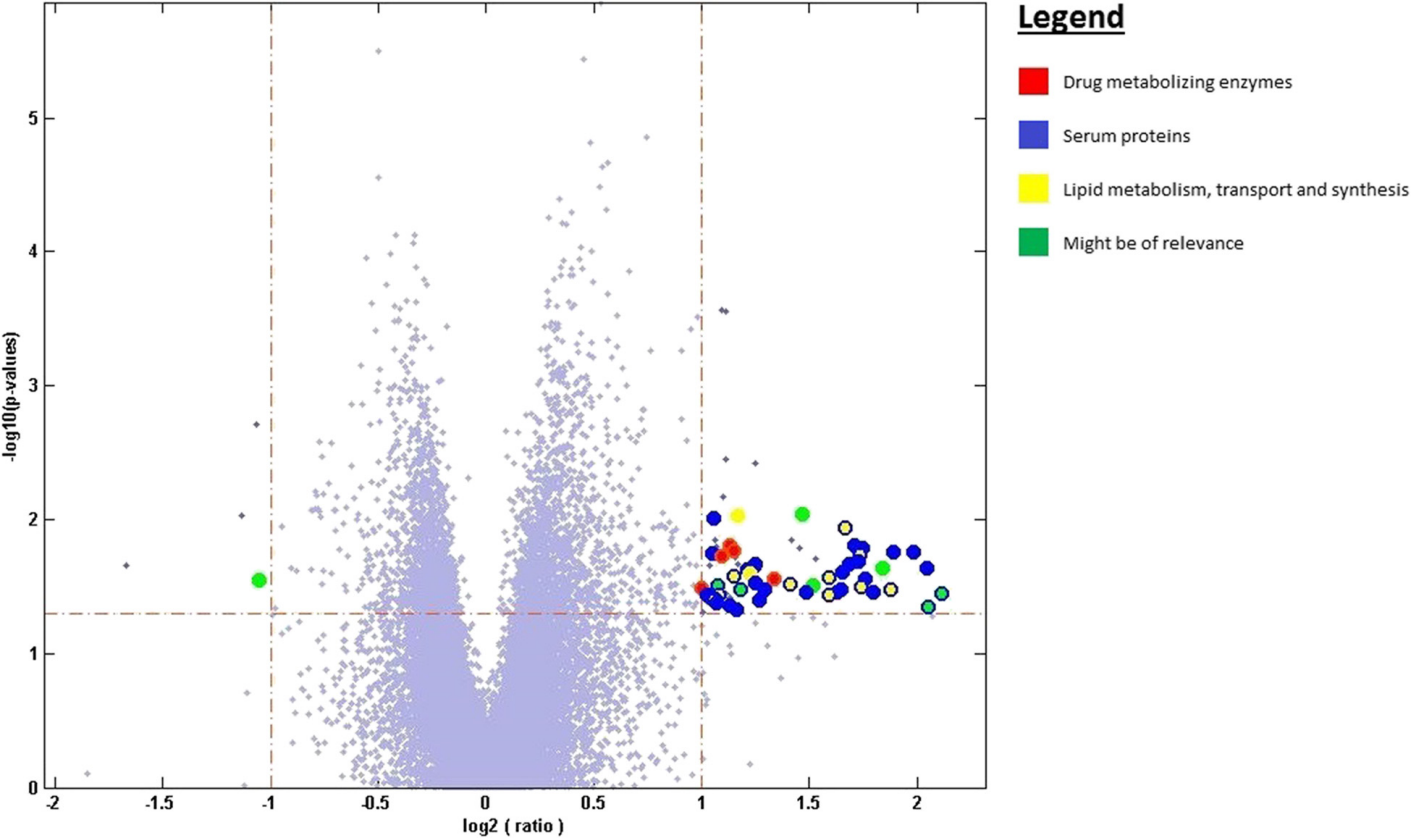
**Volcano plot illustration of the double filtering criterion.** 77 probes were significantly differentially expressed by at least 2 folds at p-value < 0.05 between responder and non-responder patients. Through Eureka-DMA's interface, genes were described and assigned to one or more of the different groups indicated.

**Figure 3:**
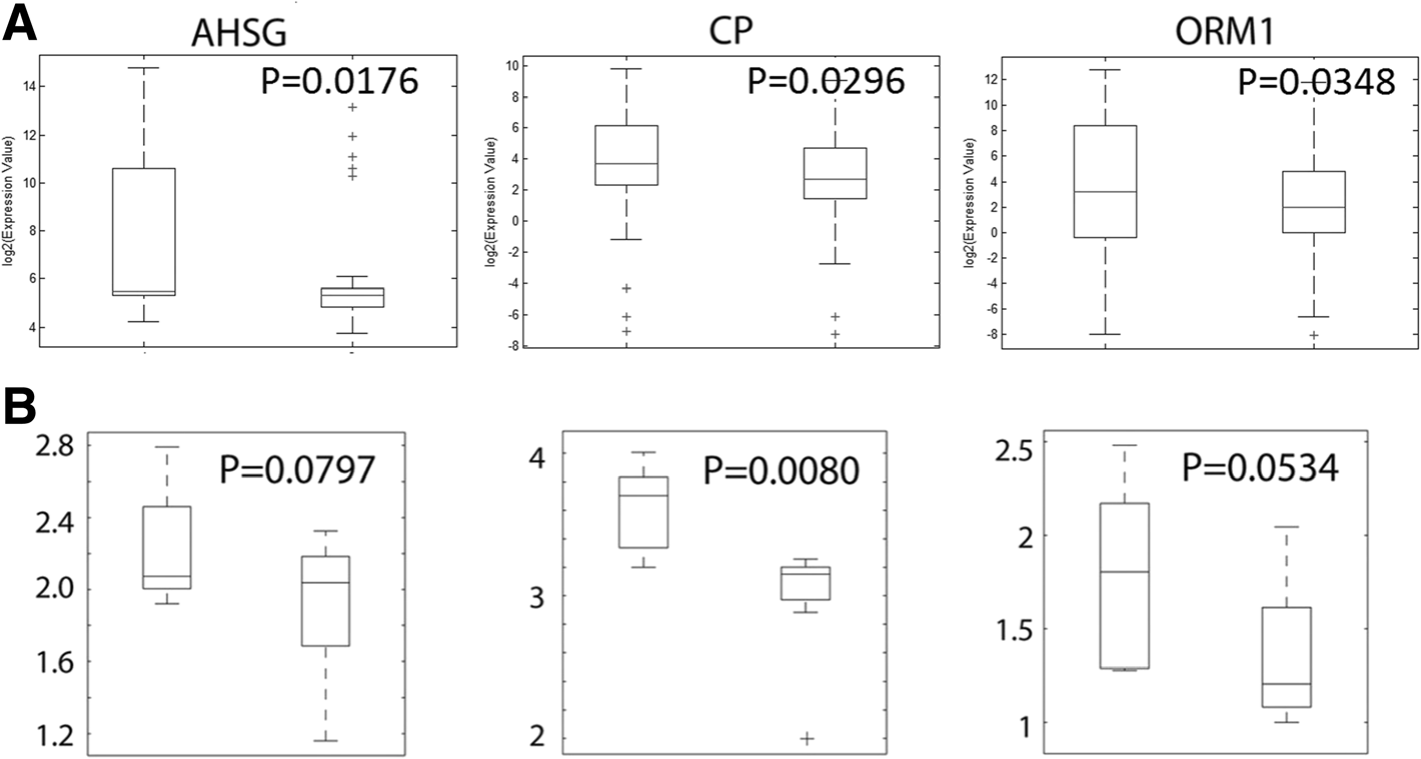
**Differential expressions of secreted proteins and theirs transcripts.** Box plots illustrations of the median and the 25th and 75th percentiles of 3 representative differentially expressed **(A)** transcripts obtained by Eureka-DMA's analysis and **(B)** proteins identified by Hyung et al. [20]. In each plot, left boxes indicate expression in the non-responders group and right boxes indicate expression in the responders group.

**Figure 4:**
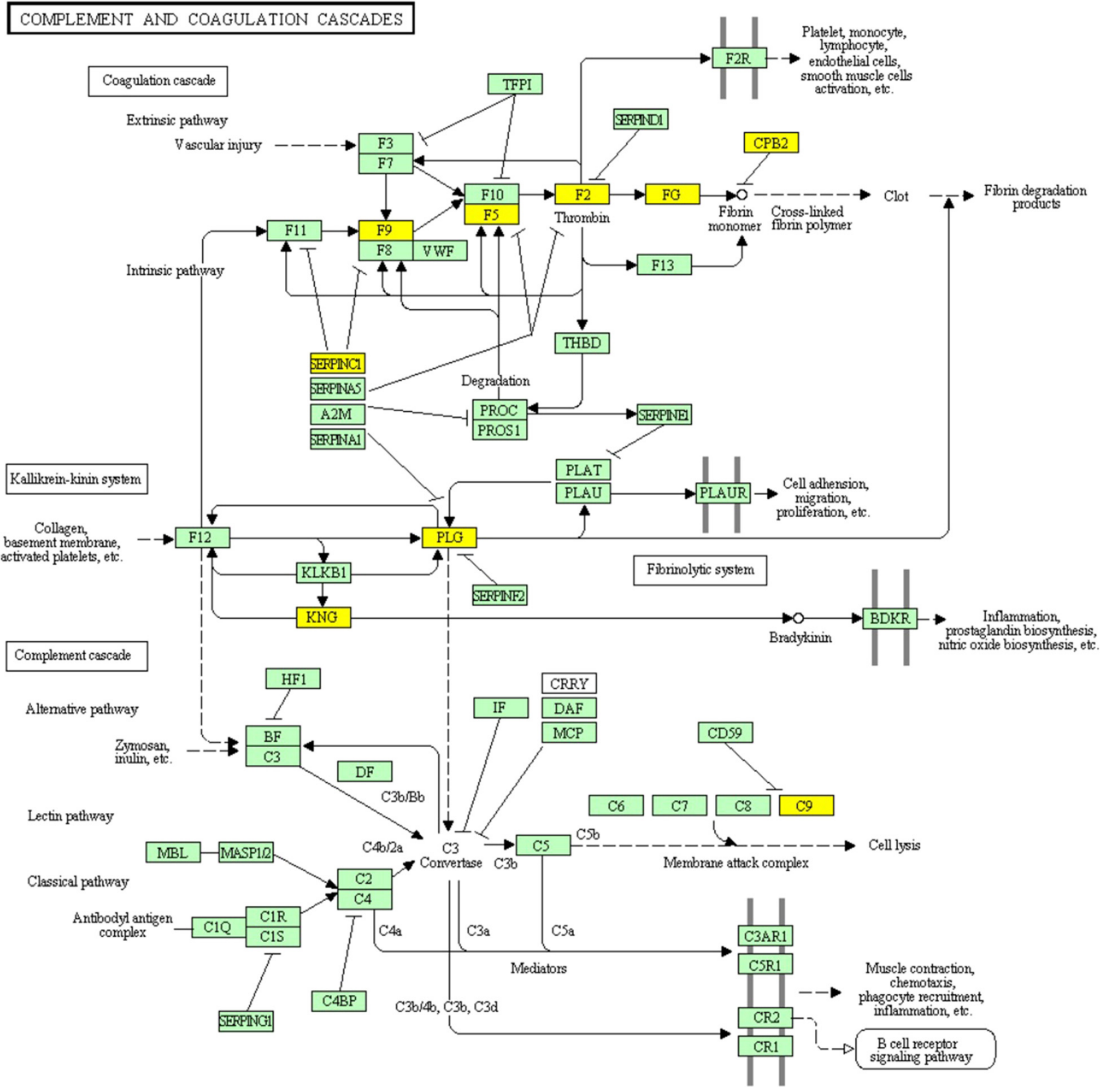
**Changes in gene expression in the complement and coagulation cascades.** Illustration of Eureka-DMA's top ranked KEGG pathway. Yellow boxes indicate genes that were found to be differentially expressed between responder and non-responder patients.

### Analysis of the lung transcriptome in mice infected with influenza A virus

**Figure 5:**
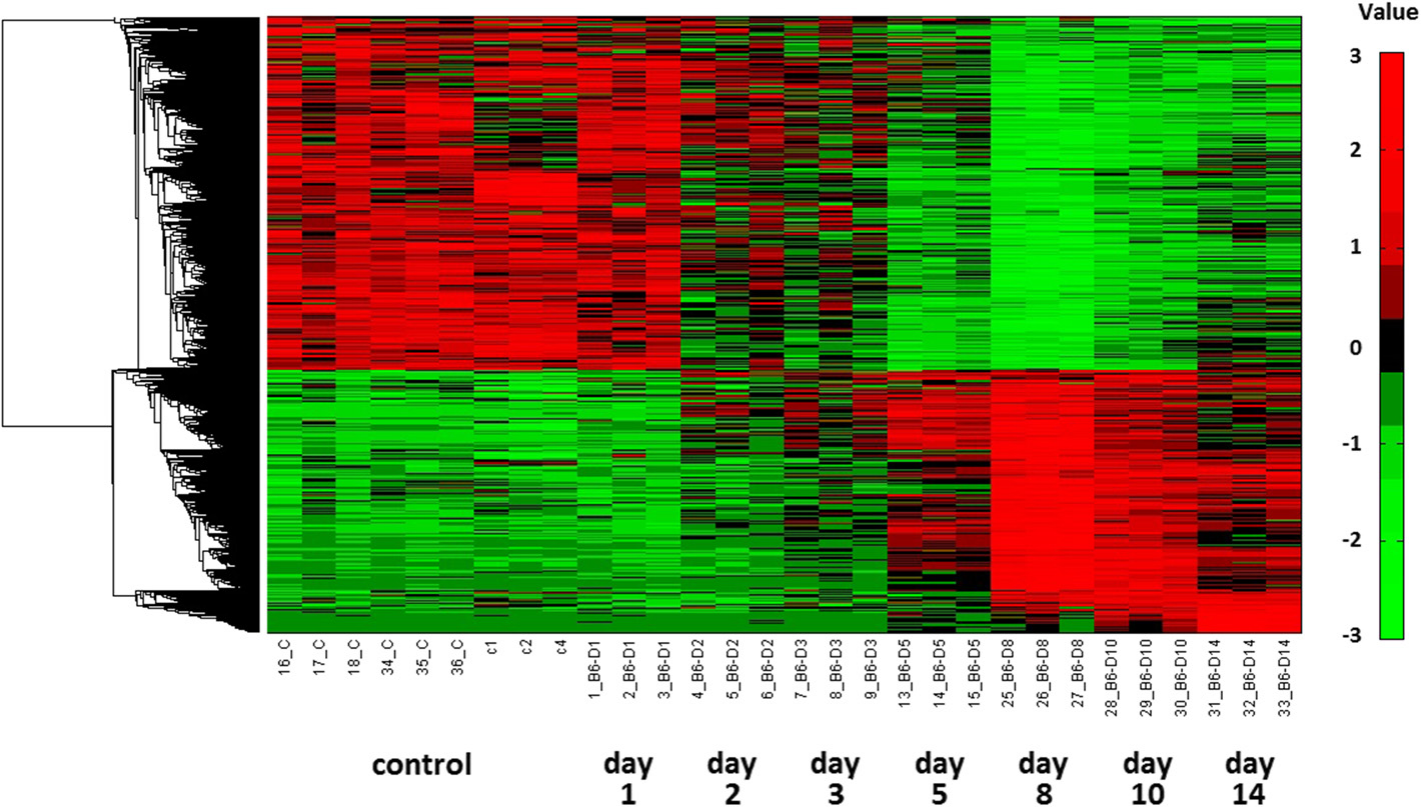
**Hierarchical clustering and heat map of genes which were found to be differentially express across 14 day's period.** 2 clusters are specified. At the top, genes that show reduced expression which starts at around day 2 and at the bottom, genes that show elevated expression which starts at around day 5. Log-transformed expression data are mean centered and hierarchically clustered by gene.

**Figure 6:**
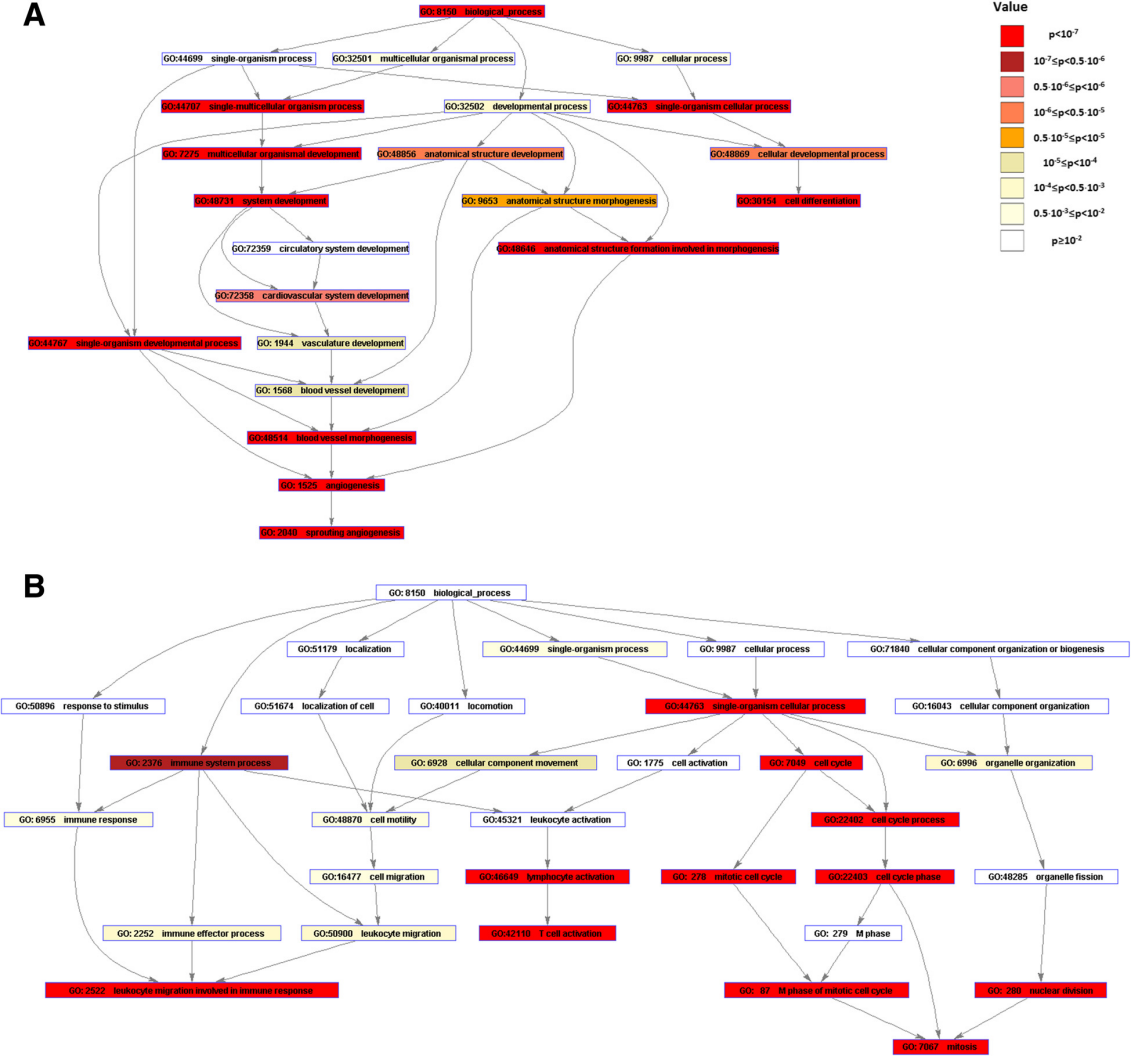
**Gene ontology structure mapping.** Hierarchical illustration of predominant gene ontology terms with respect to **(A)** the cluster of genes that demonstrate reduce expression over time and to **(B)** the cluster of genes that demonstrate elevated expression over time. Color coding reflects the terms enrichment p-value.

In order to demonstrate Eureka-DMA's credibility, we applied it to dataset published by Pommerenke et al. [26]. This study was conducted using Agilent's Whole Mouse Genome Microarray 4x44K and recorded gene expression changes in mouse lungs after a non-lethal infection with influenza A virus over a period of 60 days. Pommerenke et al. identified differentially expressed genes by comparing each group of samples, classified by the number of post infection days, to the non-infected, mice-control groups. A cutoff of two-fold change in expression levels was chosen. Density cluster analysis of all the differentially expressed genes was then used to find genes with similar expression profiles. In total, eight clusters were discernible and been subjected to an additional analysis in order to determine over-representation of gene ontology terms. Among the eight, two major clusters were observed. The first is a cluster of genes with a reduced expression starting at day 2 following infection; the expression of most of the genes in that cluster returns to baseline levels following day 14. The second cluster includes genes with an elevated expression starting at day 5, and again, following day 14 the expression of most of the genes in that cluster returns to baseline values. The two clusters were associated with developmental process, vasculature development and blood circulation GO terms and with leukocyte activation, mitosis and T cell activation GO terms respectively. The purpose of this exercise is to demonstrate the credibility of Eureka-DMA by extracting and validating results originated by different analysis tools and methodology rather than performing in-depth biological analysis of mice immune response to influenza A virus infection. Therefore, we choose to focus on part of Pommerenke et al. data, trying to derive similar biologic insights regarding genes which show reduced and increased expression across 14 day's period. The relevant quantile normalized data set was downloaded from the ArrayExpress database [5], uploaded into Eureka-DMA and the samples were categorized into their corresponding time groups by using Eureka-DMA's tool for the detection of differentially expressed genes in time series data sets. A threshold of r < 0.6 was chosen following the filtering of the 50 percent of the probes that demonstrate the lowest variance across samples. This was done in order to also expose genes which do not exhibit perfectly linear expression pattern across time and since the dataset contains uneven group sizes. The identified probes were subjected to K-means clustering which split the data into two clusters that revealed similar expression patterns to the aforementioned clusters demonstrated by Pommerenke et al. [26]. Hierarchical clustering verified the reduced and elevated expression of the genes in these clusters within the 14 days' time-frame (Figure 5). Next, we assigned the clusters with putative functional meaning and identified over-representation of gene ontology terms. Similarly to Pommerenke et al. observations, Eureka-DMA detected enrichment of the developmental process and vasculature development GO terms with several other GO terms related to ‘circulatory system’ in the gene-cluster that demonstrates reduced expression over time (Figure 6A). Furthermore, we detected enrichment of the mitosis and T cell activation GO terms with several other GO terms related to ‘leukocyte's immune response’ in the gene-cluster that demonstrates elevated expressions over time (Figure 6B). In this analysis we used different algorithms compared to Pommerenke et al. as well as a different methodology in order to extract information regarding a specific time frame. The analysis described here demonstrates Eureka-DMA's capability to validate results obtained by different tools and its credibility in the detection and interpretation of major expression patterns in highly complex datasets

## Conclusion

Eureka-DMA aspires to provide a unified and flexible platform for microarray data analysis, interpretation and visualization, and can be also used as a fast validation tool for results obtained by different analysis methods. It was programmed in MATLAB, exploiting many elements from its various toolboxes while offering friendly integration with other essential features. Eureka-DMA is the only implementation tool to provide the generation of reports that include descriptive biological information regarding genes of interests. It is also the only software for investigation and analysis of DNA microarray data to include a tool for primer design. By its novel outline, this tool allows for a quick access to the inseparable part of RTq-PCR validation that ultimately leads to the complete analysis of the data. The analysis in many other software-products is performed “behind the scene” and provides only final output; this disconnects the user from his data, potentially leading to sub-optimal decisions. Eureka-DMA unique design on the other hand, allows the user to conveniently navigate through the data and understand easily how his actions affect the outcome. Through minimal system requirements and simplicity of interface, these tools can be conveniently applied by a broader range of researchers, including biologists with limited programming or scripting skills. After testing and ensuring its capability of successfully delivering biologically-meaningful and reliable results, it is our hope that Eureka-DMA will be found useful to many other in their various research areas. Eureka-DMA's files and a step-by-step manual are freely available at the software website and are also supplemented as Additional files 3 and 4 respectively.

## Availability and requirements

**Home page:**http://blue-meduza.org/Eureka-DMA

**Operating system:** Windows, if used as a stand-alone. Application or platform independent, if used under MATLAB.

**Requirements:** If used under MATLAB: MATLAB 2010a or newer, Bioinformatics, Statistics and Image Processing Toolboxes are required. If used as a stand-alone application, MATLAB Component Runtime (MCR) is required (available to download with the software package).

**Other requirements:** Internet connection.

**License:** Free for non-commercial and academic use.

## Abbreviations

DMA: DNA microarray; GO: Gene ontology; GUI: Graphical user interface; GEO: Gene expression omnibus; PPCA: Probabilistic principal component analysis; RT-qPCR: reverse transcription quantitative PCR; CRC: Colorectal cancer; MCR: MATLAB component runtime.

## Competing interests

The authors declare that they have no competing interests.

## Authors' contributions

SA conceived the idea, designed and programmed the software and wrote the manuscript. All authors read and approved the final manuscript.

## Supplementary Material

Additional file 1 Ordered list of differentially expressed genes with theirs corresponding fold change and p-value. Click here for file

Additional file 2 Ordered list of significant enriched KEGG pathways. Click here for file

Additional file 3 **ZIP archive file that contains all the software files needed in order to run Eureka-DMA in the MATLAB environment.** This archive contains the code. Click here for file

Additional file 4 PDF file of Eureka-DMA Tutorial. Click here for file
