# Supplementary material for: Eureka-DMA: an easy-to-operate graphical user interface for fast comprehensive investigation and analysis of DNA microarray data
Source: BMC Bioinformatics. 2014 Feb 24;15:53. doi: 10.1186/1471-2105-15-53 (PMC3938137; doi:10.1186/1471-2105-15-53)
Supplement: Additional file 1 — Ordered list of differentially expressed genes with theirs corresponding fold change and p-value. [file 1471-2105-15-53-S1.pdf]

Additional file 1 Ordered list of differentially expressed genes with theirs corresponding fold change and p-value

Elevated in non-responders

Probe ID	Gene Symbol	Fold change	p-value
229596_at	AMDHD1	2.137	2.72E-04
213695_at	PON3	2.163	2.78E-04
238625_at	C1orf168	2.158	3.52E-03
235947_at	AA922273	2.383	3.78E-03
228494_at	PPP1R9A	2.142	6.76E-03
224367_at	BEX2	2.768	9.03E-03
206340_at	NR1H4	2.249	9.39E-03
231029_at	F5	2.077	9.80E-03
205969_at	AADAC	3.202	1.10E-02
205108_s_at	APOB	3.177	1.15E-02
223721_s_at	DNAJC12	2.675	1.42E-02
218976_at	DNAJC12	2.086	1.42E-02
214421_x_at	CYP2C9	2.194	1.55E-02
205650_s_at	FGA	3.266	1.56E-02
210751_s_at	RGN	2.101	1.59E-02
203400_s_at	TF	3.364	1.61E-02
205719_s_at	PAH	2.742	1.61E-02
40665_at	FMO3	2.215	1.71E-02
219612_s_at	FGG	3.959	1.76E-02
210929_s_at	AHSG	3.710	1.76E-02
217512_at	KNG1	2.071	1.77E-02
217073_x_at	APOA1	3.330	1.82E-02
221605_s_at	PIPOX	2.172	1.82E-02
220017_x_at	CYP2C9	2.132	1.87E-02
244524_at	AI587332	2.123	1.90E-02
204987_at	ITIH2	2.227	1.91E-02
206505_at	UGT2B4	2.889	1.98E-02
204988_at	FGB	3.319	2.06E-02
204534_at	VTN	3.212	2.13E-02
206054_at	KNG1	2.380	2.13E-02
205422_s_at	ITGBL1	2.245	2.16E-02
206177_s_at	ARG1	2.057	2.19E-02
214063_s_at	TF	2.366	2.22E-02
208470_s_at	HPR	4.126	2.30E-02
205302_at	IGFBP1	3.583	2.32E-02
205754_at	F2	2.319	2.35E-02
206697_s_at	HP	3.148	2.49E-02
238441_at	PRKAA2	2.342	2.54E-02
205477_s_at	AMBP	2.223	2.65E-02
206651_s_at	CPB2	2.531	2.69E-02
205216_s_at	APOH	3.013	2.72E-02
209976_s_at	CYP2E1	2.524	2.74E-02
205649_s_at	FGA	3.382	2.74E-02
204846_at	CP	2.376	2.96E-02
205820_s_at	APOC3	2.666	3.05E-02
206350_at	APCS	2.860	3.07E-02
214842_s_at	ALB	2.108	3.12E-02
219465_at	APOA2	3.342	3.18E-02
208147_s_at	CYP2C8	2.002	3.24E-02
206226_at	HRG	2.270	3.29E-02
219466_s_at	APOA2	3.684	3.31E-02
⋮			
205305_at	FGL1	2.410	3.33E-02
206727_at	C9	2.452	3.34E-02
204965_at	GC	3.129	3.34E-02
216238_s_at	FGB	2.799	3.44E-02
205041_s_at	ORM2	3.100	3.46E-02
205040_at	ORM1	3.481	3.48E-02
206535_at	SLC2A2	2.164	3.52E-02
211298_s_at	ALB	4.339	3.56E-02
220148_at	ALDH8A1	2.135	3.64E-02
210049_at	SERPINC1	2.035	3.66E-02
214461_at	LBP	3.017	3.67E-02
204450_x_at	APOA1	2.116	3.67E-02
207409_at	LECT2	2.188	3.82E-02
1558034_s_at	CP	2.089	3.93E-02
207218_at	F9	2.412	3.96E-02
210445_at	FABP6	2.084	4.01E-02
206024_at	HPD	2.035	4.14E-02
205755_at	ITIH3	2.094	4.18E-02
214456_x_at	SAA2	2.191	4.37E-02
1565228_s_at	ALB	4.143	4.52E-02
209977_at	PLG	2.240	4.70E-02
206287_s_at	MUSTN1	2.012	4.88E-02

Elevated in responders

225767_at	LOC284801	2.094	1.95E-03
225762_x_at	LOC284801	2.195	9.40E-03
213479_at	NPTX2	3.181	2.21E-02
200951_s_at	CCND2	2.077	2.81E-02

Legend

*** Knowledge based on Eureka's Gene description tool alone**

- Drug metabolizing enzymes
- Serum proteins
- Lipid metabolism, transport and synthesis
- Might be of relevance

*** Also found as classifiers at Seok-Won Hyung et al.**

Thresholds

*p- value ≤ 0.05

*fold change ≥2
