# Supplementary material for: Eureka-DMA: an easy-to-operate graphical user interface for fast comprehensive investigation and analysis of DNA microarray data
Source: BMC Bioinformatics. 2014 Feb 24;15:53. doi: 10.1186/1471-2105-15-53 (PMC3938137; doi:10.1186/1471-2105-15-53)
Supplement: Additional file 2 — Ordered list of significant enriched KEGG pathways. [file 1471-2105-15-53-S2.pdf]

Additional file 2 Ordered list of significant enriched KEGG pathways

KEGG pathway ID	Pathway name	p-value
hsa04610	Complement and coagulation cascades - Homo sapiens (human)	7.19E-14
hsa00982	Drug metabolism - cytochrome P450 - Homo sapiens (human)	5.67E-05
hsa03320	PPAR signaling pathway - Homo sapiens (human)	6.61E-04
hsa00591	Linoleic acid metabolism - Homo sapiens (human)	8.79E-04
hsa00980	Metabolism of xenobiotics by cytochrome P450 - Homo sapiens (human)	1.07E-03
hsa05204	Chemical carcinogenesis - Homo sapiens (human)	1.16E-03
hsa00360	Phenylalanine metabolism - Homo sapiens (human)	4.98E-03
hsa00590	Arachidonic acid metabolism - Homo sapiens (human)	6.15E-03
hsa00830	Retinol metabolism - Homo sapiens (human)	6.15E-03
hsa04977	Vitamin digestion and absorption - Homo sapiens (human)	8.45E-03
hsa00053	Ascorbate and aldarate metabolism - Homo sapiens (human)	1.05E-02
hsa05143	African trypanosomiasis - Homo sapiens (human)	1.59E-02
hsa04975	Fat digestion and absorption - Homo sapiens (human)	2.21E-02
hsa00860	Porphyrin and chlorophyll metabolism - Homo sapiens (human)	2.40E-02
hsa00400	Phenylalanine, tyrosine and tryptophan biosynthesis - Homo sapiens (human)	3.34E-02
hsa01100	Metabolic pathways - Homo sapiens (human)	3.47E-02
hsa05150	Staphylococcus aureus infection - Homo sapiens (human)	4.12E-02
