# Supplementary material for: Eureka-DMA: an easy-to-operate graphical user interface for fast comprehensive investigation and analysis of DNA microarray data
Source: BMC Bioinformatics. 2014 Feb 24;15:53. doi: 10.1186/1471-2105-15-53 (PMC3938137; doi:10.1186/1471-2105-15-53)
Supplement: Additional file 3 — ZIP archive file that contains all the software files needed in order to run Eureka-DMA in the MATLAB environment. This archive contains the code. [file 1471-2105-15-53-S3.zip › Eureka-DMA/Eureka-DMA Tutorial.pdf]

Eureka-DMA – Tutorial

1. MATLAB

If you are not intending to use the windows standalone version of Eureka-DMA, please be sure that you are using an original copy of Matlab® and set the path to Eureka-DMA directory as describe in the picture.
If you are using different version of MATLAB, it might be done a little bit differently.

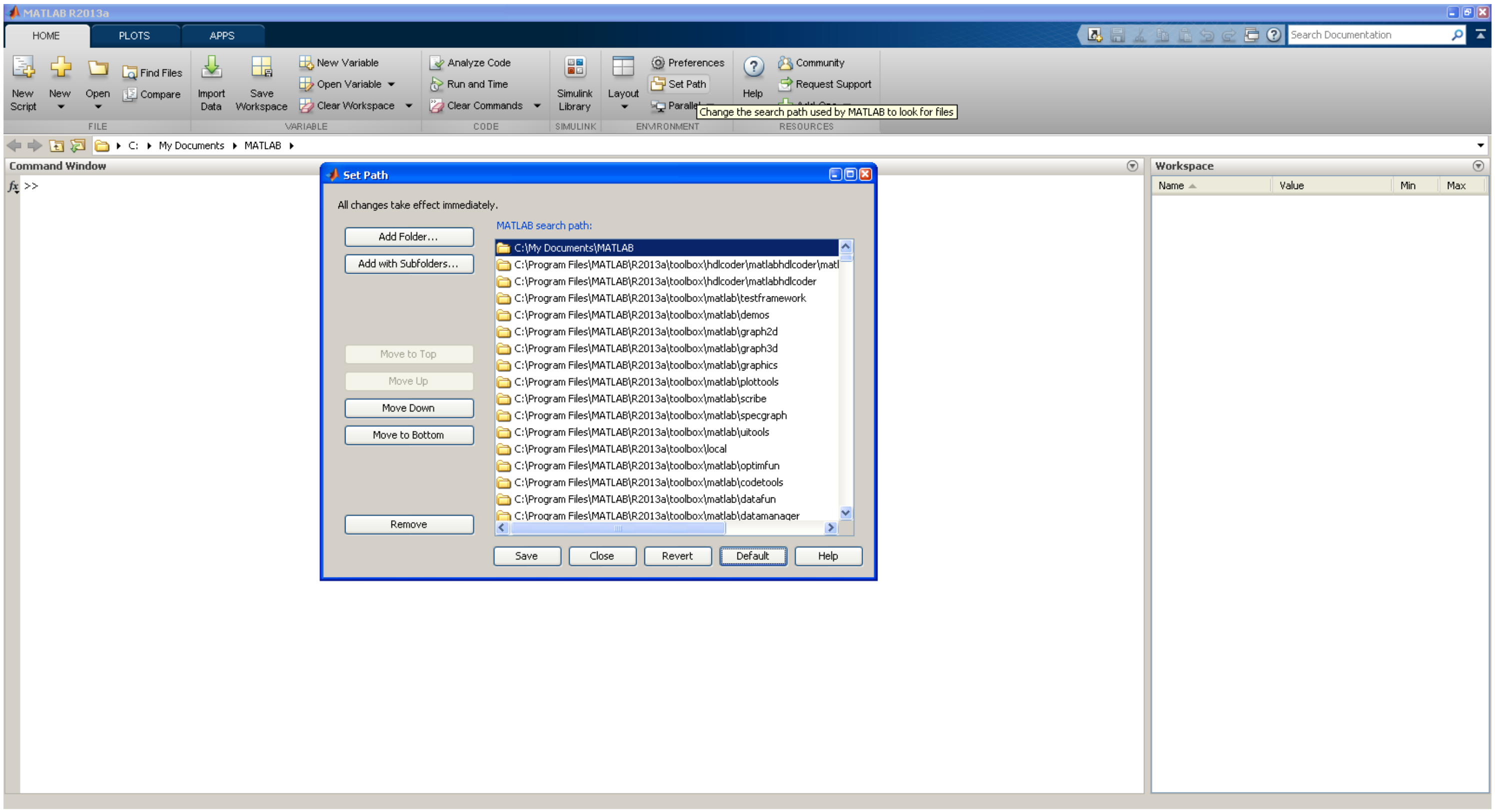

Once Matlab is set, type Eureka_DMA in the command window to get it going.

2. Edit the raw data file

Eureka-DMA originally developed to analyze DNA microarray data (Illumina, Affymetrix, etc.) but other data types can also be analyzed to some extent with appropriate editing.

In order to start working on your data please check that it's organized as describe in the pictures.

This configuration can be easily obtained by exportation from the various microarray manufacturer company's softwares. as txt or windows Excel files.

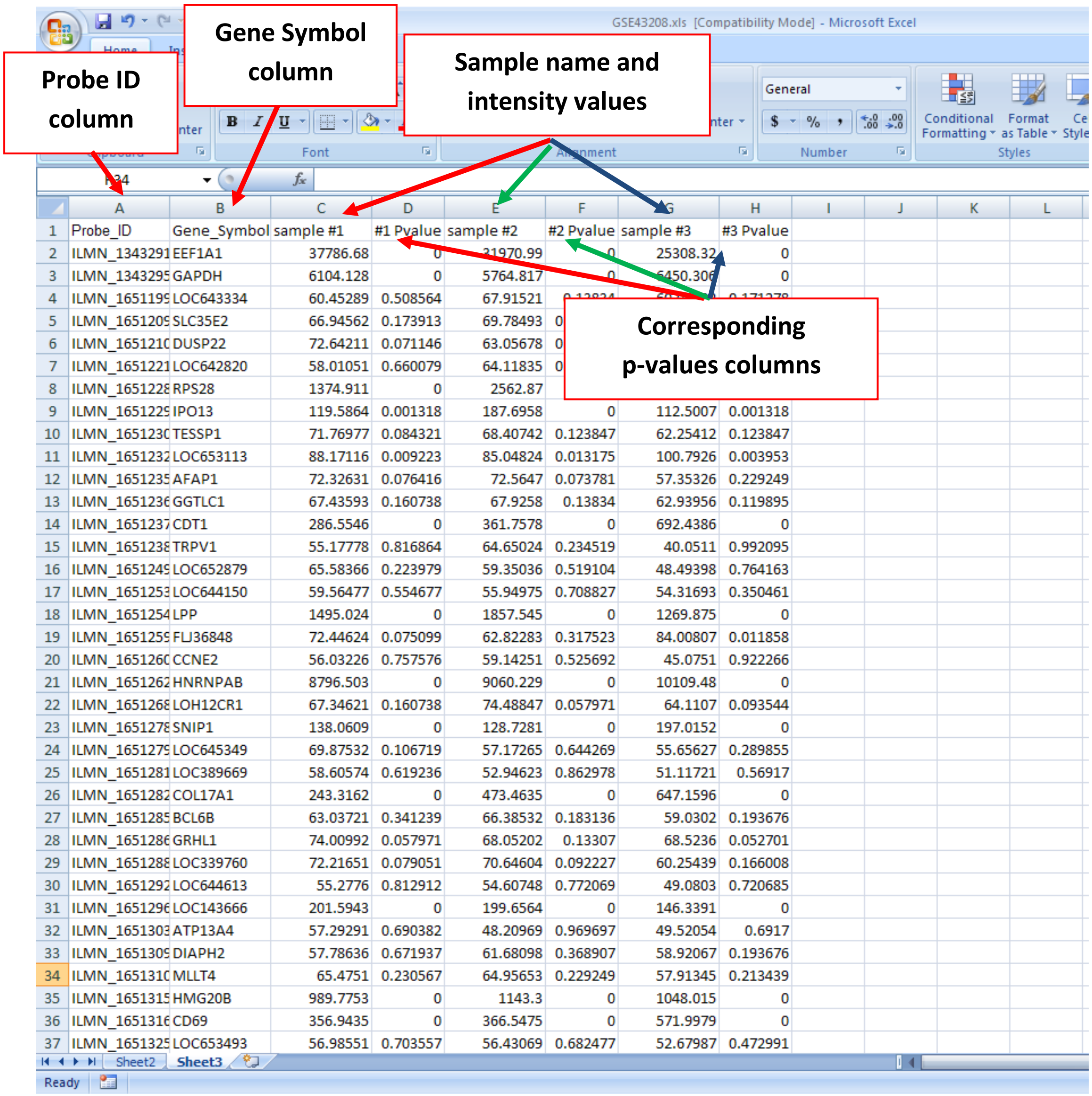

	A	B	C	D	E	F	G	H
1	Probe_ID	Gene_Symbol	sample #1	#1 Pvalue	sample #2	#2 Pvalue	sample #3	#3 Pvalue
2	ILMN_1343291	EEF1A1	37786.68	0	31970.99	0	25308.32	0
3	ILMN_1343295	GAPDH	6104.128	0	5764.817	0	6450.306	0
4	ILMN_1651199	LOC643334	60.45289	0.508564	67.91521	[illegible]	[illegible]	[illegible]
5	ILMN_1651209	SLC35E2	66.94562	0.173913	69.78493	[illegible]	[illegible]	[illegible]
6	ILMN_1651210	DUSP22	72.64211	0.071146	63.05678	[illegible]	[illegible]	[illegible]
7	ILMN_1651221	LOC642820	58.01051	0.660079	64.11835	[illegible]	[illegible]	[illegible]
8	ILMN_1651228	RPS28	1374.911	0	2562.87	[illegible]	[illegible]	[illegible]
9	ILMN_1651229	IPO13	119.5864	0.001318	187.6958	0	112.5007	0.001318
10	ILMN_1651230	TESSP1	71.76977	0.084321	68.40742	0.123847	62.25412	0.123847
11	ILMN_1651232	LOC653113	88.17116	0.009223	85.04824	0.013175	100.7926	0.003953
12	ILMN_1651235	AFAP1	72.32631	0.076416	72.5647	0.073781	57.35326	0.229249
13	ILMN_1651236	GGTLC1	67.43593	0.160738	67.9258	0.13834	62.93956	0.119895
14	ILMN_1651237	CDT1	286.5546	0	361.7578	0	692.4386	0
15	ILMN_1651238	TRPV1	55.17778	0.816864	64.65024	0.234519	40.0511	0.992095
16	ILMN_1651249	LOC652879	65.58366	0.223979	59.35036	0.519104	48.49398	0.764163
17	ILMN_1651253	LOC644150	59.56477	0.554677	55.94975	0.708827	54.31693	0.350461
18	ILMN_1651254	LPP	1495.024	0	1857.545	0	1269.875	0
19	ILMN_1651259	FLJ36848	72.44624	0.075099	62.82283	0.317523	84.00807	0.011858
20	ILMN_1651260	CCNE2	56.03226	0.757576	59.14251	0.525692	45.0751	0.922266
21	ILMN_1651262	HNRNPAB	8796.503	0	9060.229	0	10109.48	0
22	ILMN_1651268	LOH12CR1	67.34621	0.160738	74.48847	0.057971	64.1107	0.093544
23	ILMN_1651278	SNIP1	138.0609	0	128.7281	0	197.0152	0
24	ILMN_1651279	LOC645349	69.87532	0.106719	57.17265	0.644269	55.65627	0.289855
25	ILMN_1651281	LOC389669	58.60574	0.619236	52.94623	0.862978	51.11721	0.56917
26	ILMN_1651282	COL17A1	243.3162	0	473.4635	0	647.1596	0
27	ILMN_1651285	BCL6B	63.03721	0.341239	66.38532	0.183136	59.0302	0.193676
28	ILMN_1651286	GRHL1	74.00992	0.057971	68.05202	0.13307	68.5236	0.052701
29	ILMN_1651288	LOC339760	72.21651	0.079051	70.64604	0.092227	60.25439	0.166008
30	ILMN_1651292	LOC644613	55.2776	0.812912	54.60748	0.772069	49.0803	0.720685
31	ILMN_1651296	LOC143666	201.5943	0	199.6564	0	146.3391	0
32	ILMN_1651303	ATP13A4	57.29291	0.690382	48.20969	0.969697	49.52054	0.6917
33	ILMN_1651309	DIAPH2	57.78636	0.671937	61.68098	0.368907	58.92067	0.193676
34	ILMN_1651310	MLLT4	65.4751	0.230567	64.95653	0.229249	57.91345	0.213439
35	ILMN_1651315	HMG20B	989.7753	0	1143.3	0	1048.015	0
36	ILMN_1651316	CD69	356.9435	0	366.5475	0	571.9979	0
37	ILMN_1651325	LOC653493	56.98551	0.703557	56.43069	0.682477	52.67987	0.472991

If you do not have information about detection p-values just omit the p-value columns. (it is highly recommended to work with detection p-values so try to ask for the full raw data from your microarray service suppliers).

Save the file in 'xls', 'xlsx' or 'txt' format.

3.Eureka-DMA's graphical user interfaces (GUIs)

*please notice that Eureka-DMA's GUIs looks best on resolution of 1600x900 pixels

3.1. **Upload and export data /Box plot:** Load your file and start exploring the data by viewing the Box plot

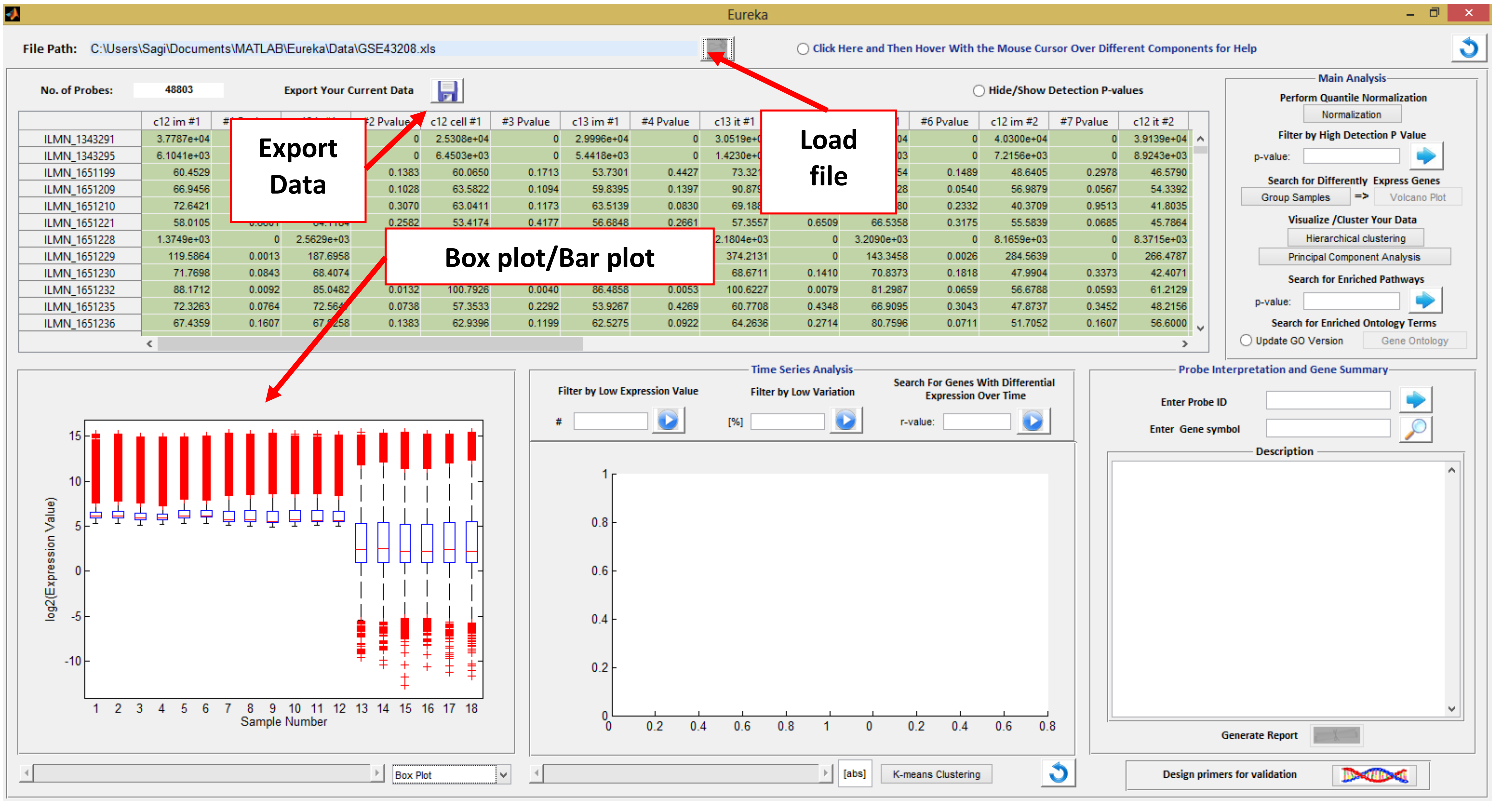

The Box plot is very useful for comparing distributions between the different samples. The plot contains the maximum and minimum values in each sample, the lower and upper quartiles, and the median. In this example you can see that all the last six samples (the data for these samples originally obtained from different Bead-chip array) has significant lower intensity values than the first twelve samples.

By pushing the export data button, you can export the data shown on the table at any time during the analysis and save it in 'xls', 'xlsx' or 'txt' formats for further processing with other external tools.

3.2. Help button: if it's your first time using Eureka-DMA, we recommend using the help button and get to know Eureka-DMA's components and functions

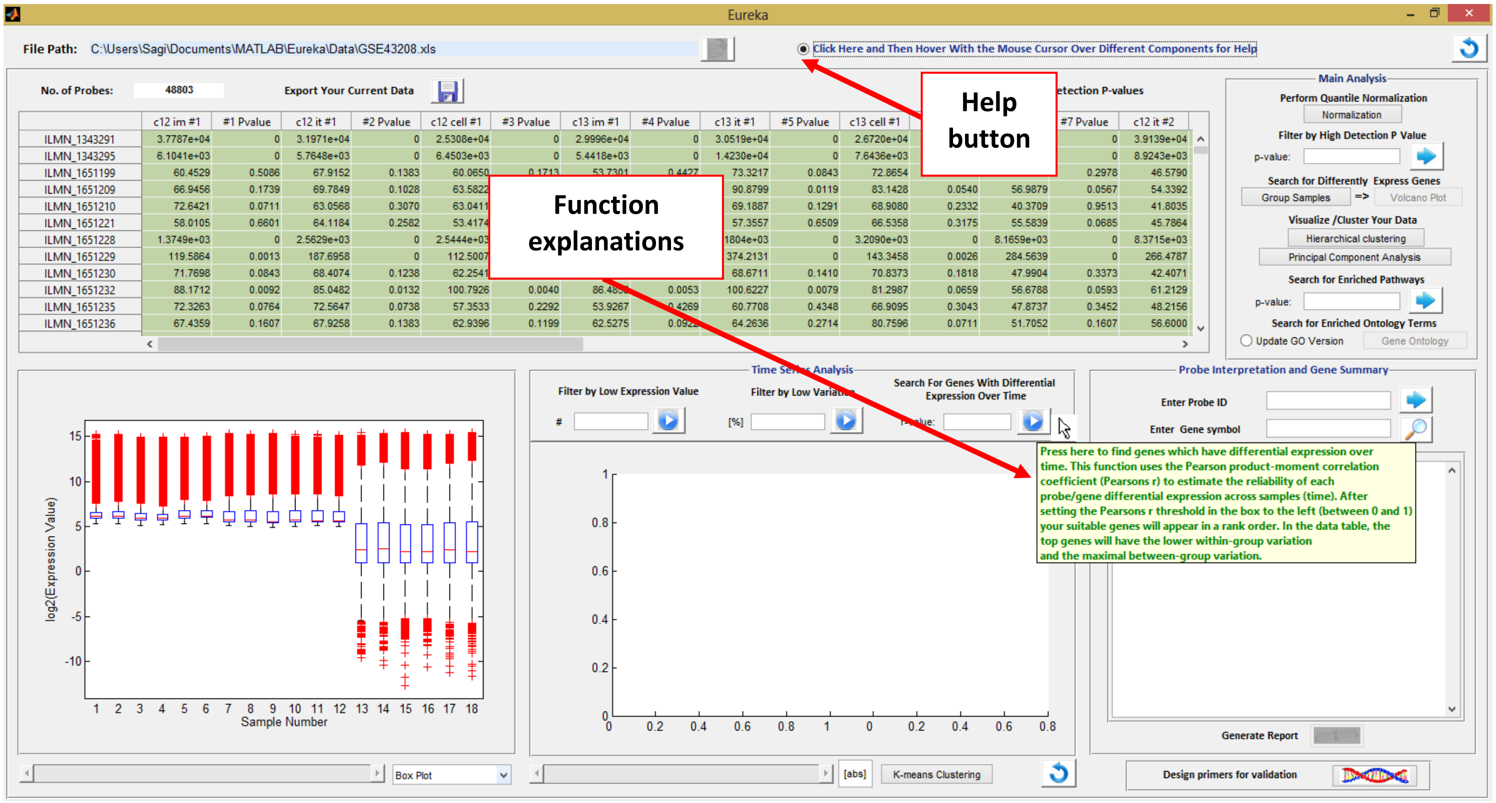

3.2. Normalization: In order to avoid systematic variation of non-biological origin and to ensure that differences in intensities are indeed due to differential expression; you will need to perform normalization. Eureka-DMA performs quantile normalization, a method which is frequently used in microarray data analysis. You can see that after normalization the intensities' distributions across all the samples are identical

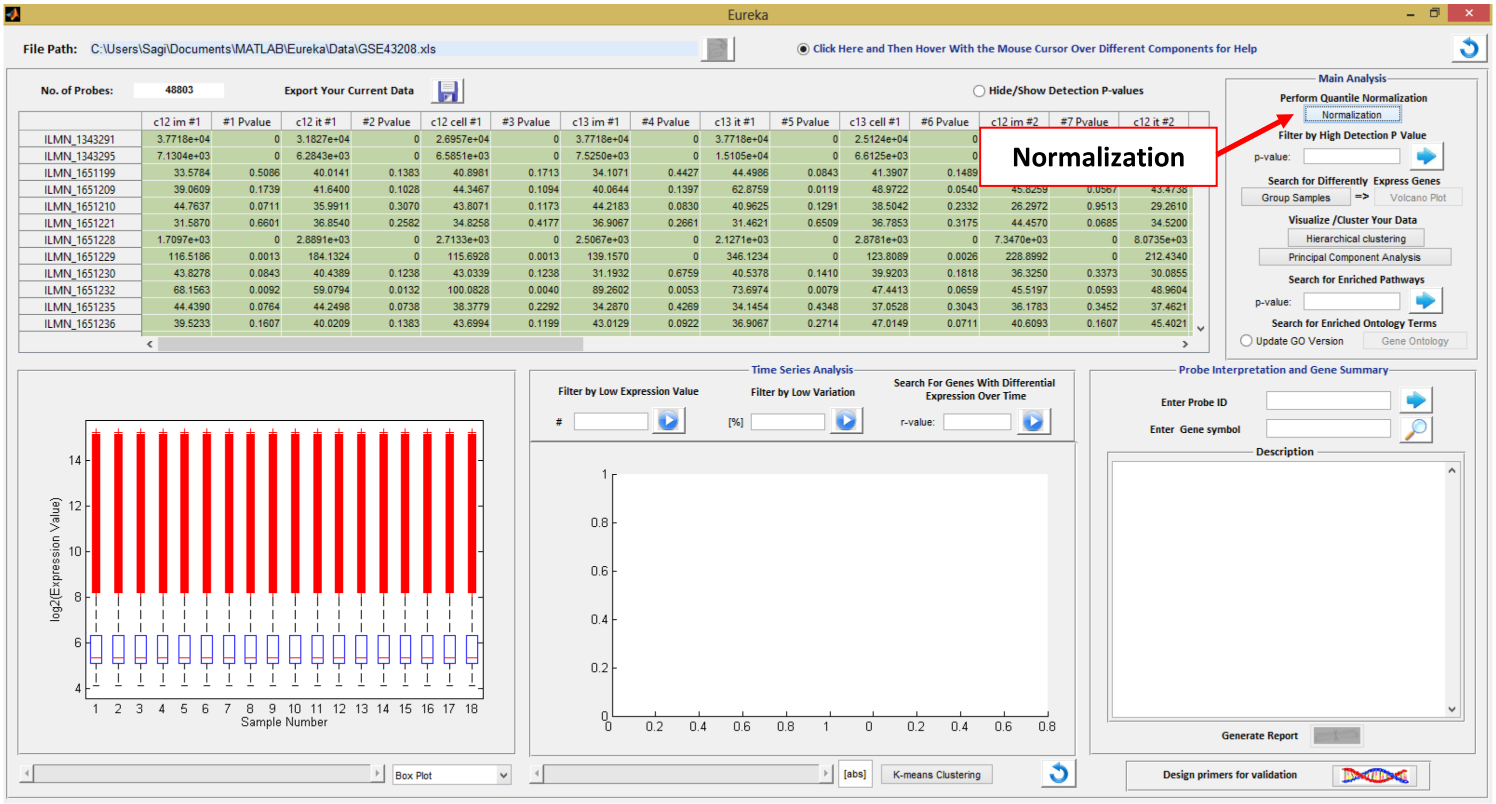

3.3. Bar plot: you can switch to bar plot view in order to observe the expression values of the different probes/genes across the samples. Use the slider to navigate between them.

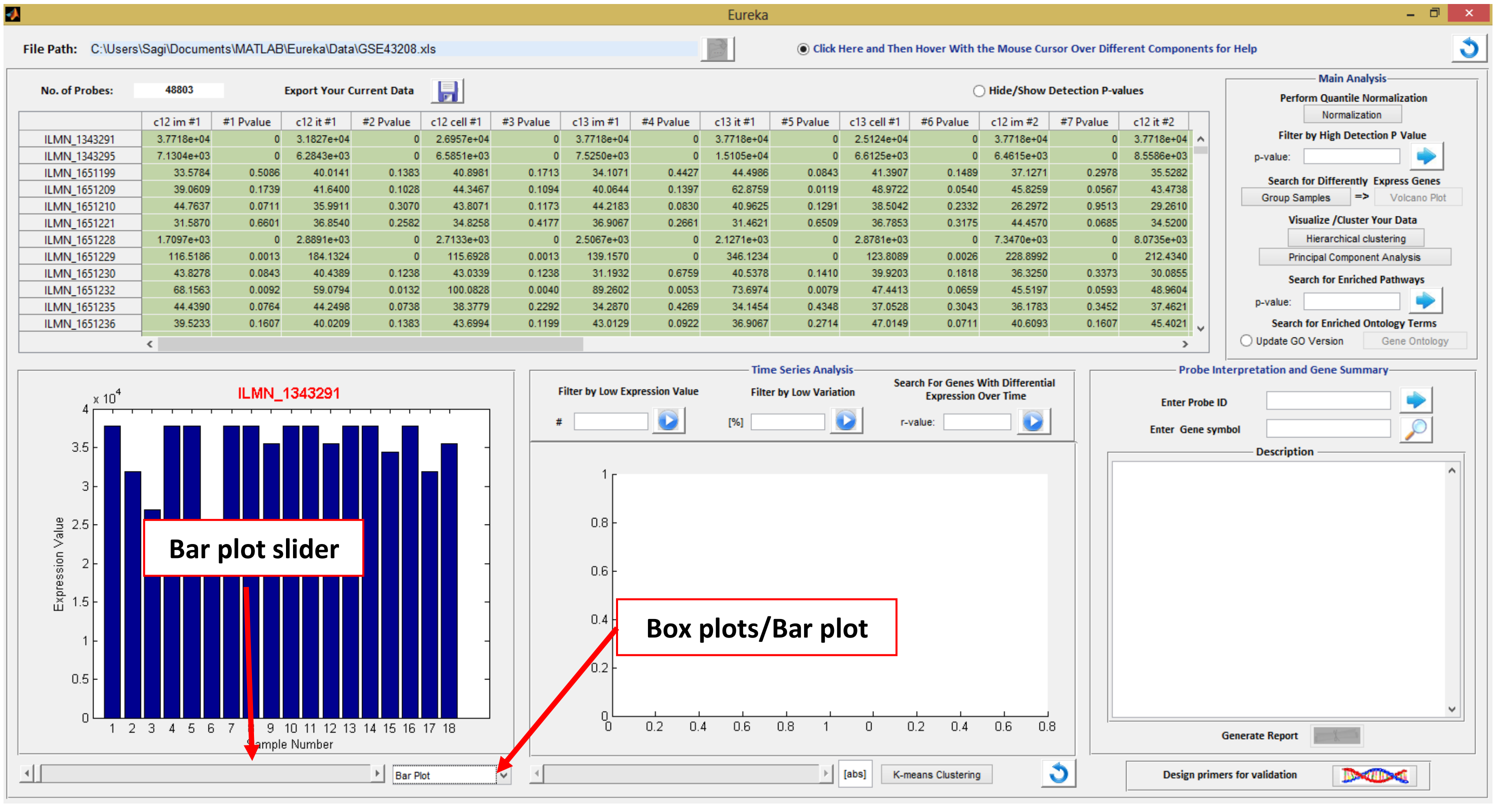

3.4 p-value filter/ Low value filter: it is most likely that your data contain genes that are not expressed in any of your samples and therefore are not relevant for further analysis.

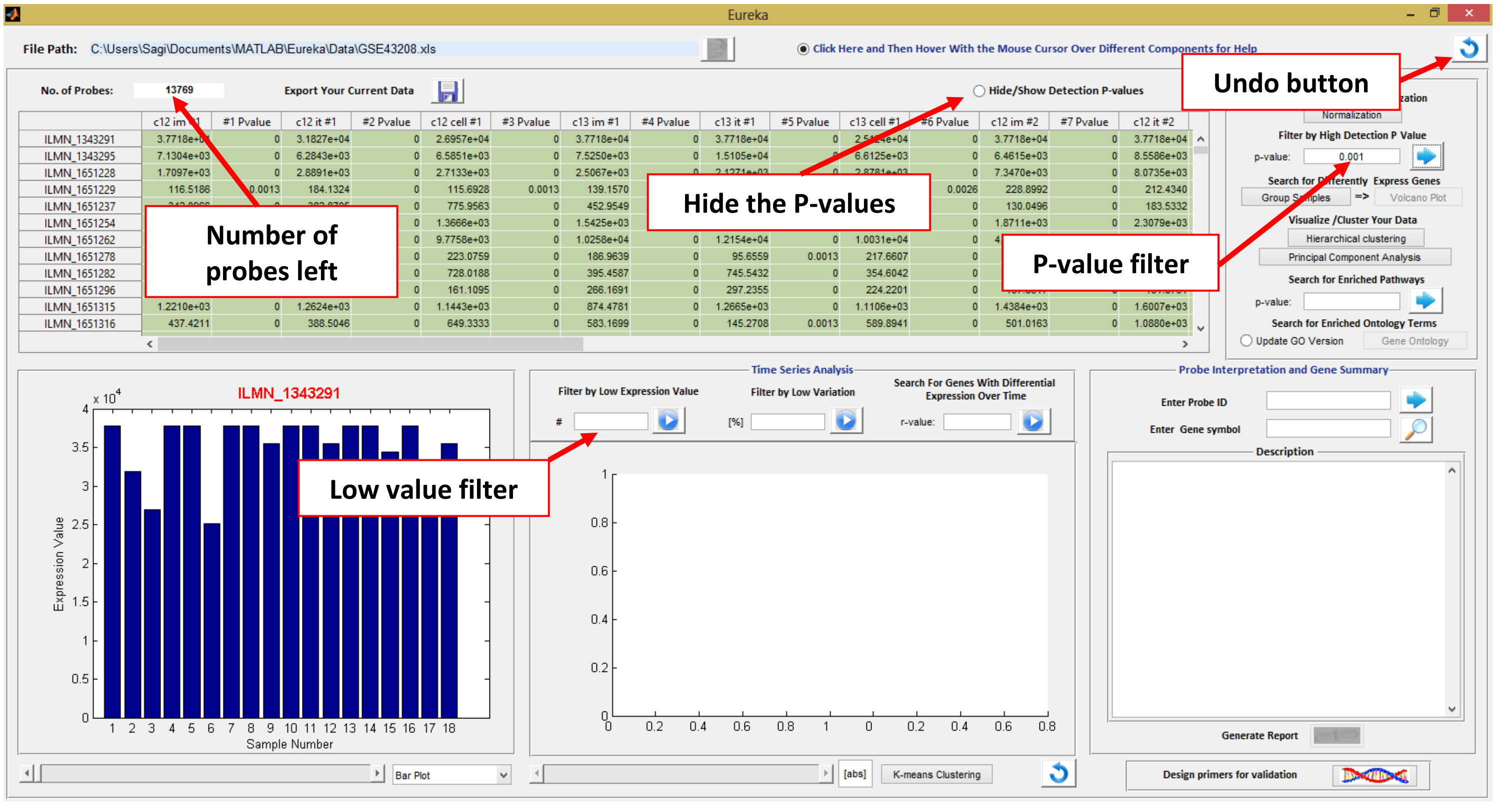

Set p-value threshold (generally bellow 0.01) and execute the filter in order to delete probes which do not have higher intensity values than the background defined by the chip-array internal negative control probes. You will also see that the window that indicates the number of probes in your current data will be updated after executing the filter. Keep tracking the number of entries as you further continue with the analysis.
If your data does not contains detection p-values you can minimize the content of the irrelevant probes/genes by using the Low value filter. Execute that filter to delete probes which do not have higher intensity values than your defined threshold. You can estimate the threshold by looking at signal intensities of genes that you know that are not expressed in your samples (for example, if your sample's origin is kidneys, you will not expect to find genes which are only expressed in brain tissue). You can also get estimation by looking on the signals of the internal negative controls. Be careful not to filter too much data. If you think you did, you can always use the Undo button.
When all set and done, you can hide the p-value columns by clicking on the appropriate button.

3.5 Find differentially express genes/ assign groups for your samples:

In order to find differentially expressed genes you will need to categorize your samples into one of two groups. After pressing the 'Group Samples' button, a new window will appear

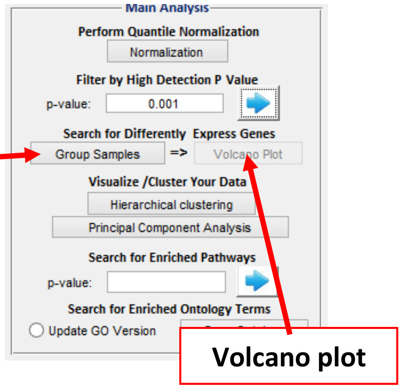

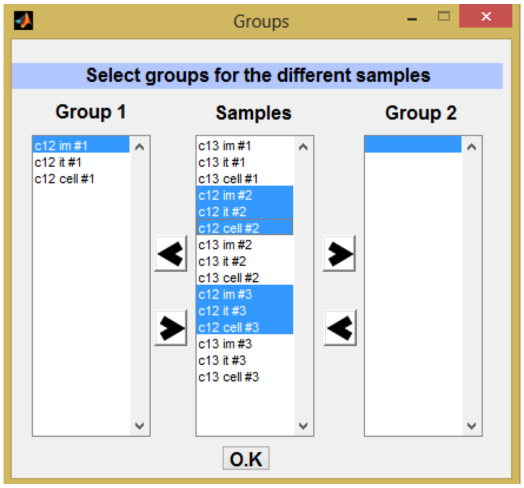

Move your samples into the boxes that indicate the different groups. Please notice that you can choose to analyze only part of your samples and not to continue with all of them.
After doing so, press O.K. and create the Volcano plot by pressing on the appropriate button on Eureka-DMA's main window

3.6 Find differentially expressed genes/ Volcano plot:

The X-axis of the volcano plot corresponds to log2 (fold change) of the differentially expressed gene's intensity between the groups while the values on the Y-axis corresponds to –log10 (p-value). The p-value is determined by two tailed t-test and it estimates the statistical signification of the fold change.

You can see in the right windows the Probe IDs and the name of the differentially expressed genes, how much their expression is changed between the groups and the matching p-value.

*The p-values are not adjusted for multiple comparisons but you can simply multiply the values with the number of probes that were in your data before executing the volcano plot function (Bonferroni correction).

You can mark one or more genes to find where they appear on the volcano plot. You can also copy the data directly from here to other software that allow copy/paste . Press the Clear button to clear your selection.

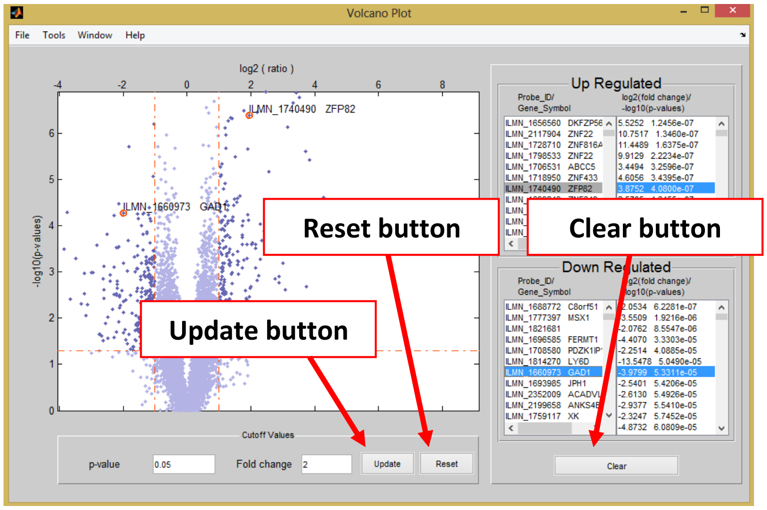

The initial p-value and fold change cutoffs are set to 0.05 and 1.5 respectively but you can change them within the Volcano plot GUI.
Afterward, press the Update button to update the data table and the number of probes in Eureka-DMA's main GUI.
Press the Reset button in order to reset into the initial cutoffs.

Please notice that now your samples re-ordered, first, to the left, the samples belong to group 1 and after them the samples belong to group 2. Samples which do not been assign to either one of the groups will be omitted from the data table and will not included in further analyses.

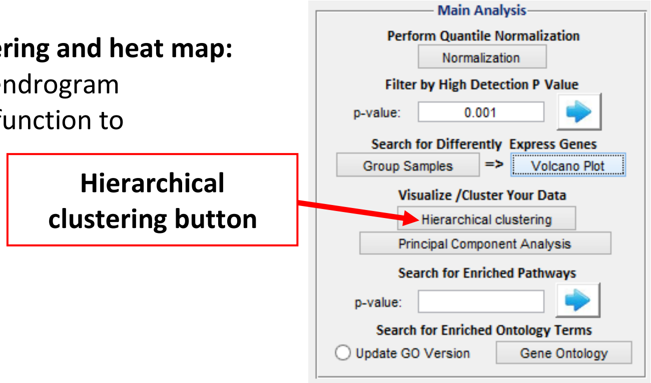

3.7 Explore the differentially expressed genes/ hierarchical clustering and heat map:

By pressing the hierarchical clustering button you will create dendrogram and heat map. Please notice that if you are trying to apply this function to More than 5000 probes you will get that warning notice.

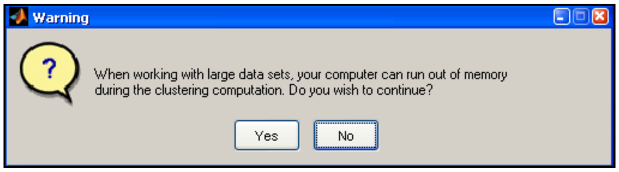

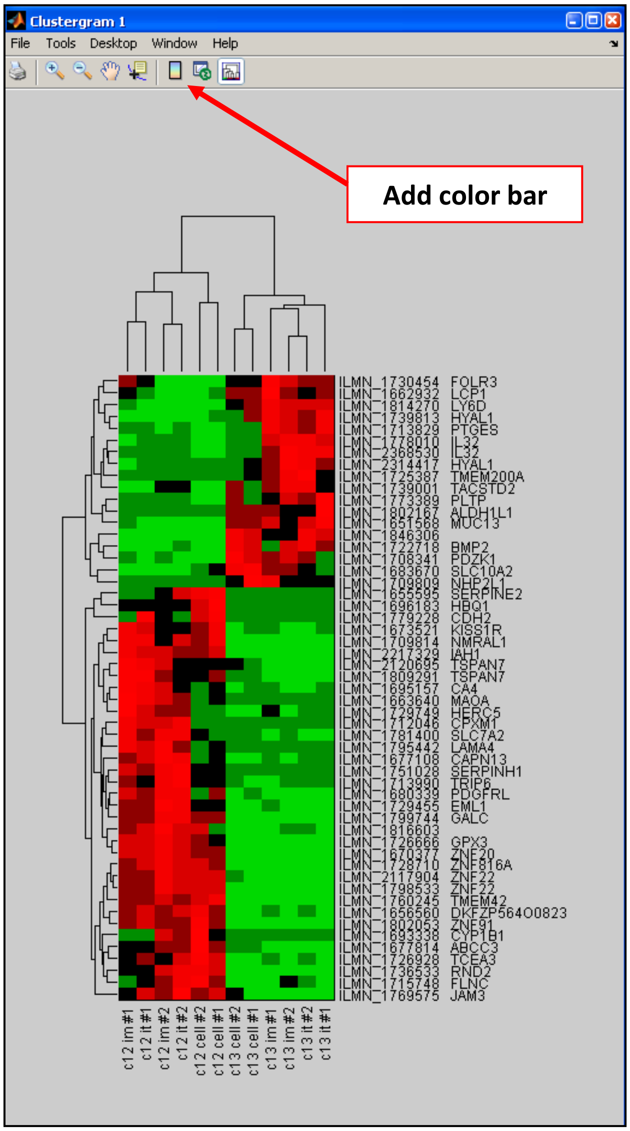

<u>The heat map</u>: the heat map demonstrates the expression of genes in each sample. Red colors indicate high expression and green colors indicate low expression. The color is chosen by transforming the intensity values so that the mean is zero and the standard deviation is one for each gene across all the samples. You can add color bar by pressing on the appropriate button. The top and left sided dendrograms represent the similarities between samples and between genes respectively. The closest the distance between two dendrograms lines, the greater the similarity between the two samples or genes in respect to their expression across columns or across rows.
The distance metric to calculate the pairwise distances between genes and between samples is Euclidean and the linkage method is unweighted average distance (UPGMA)

*when analyzing time series data, only gene clustering will be preformed

3.7 **Probabilistic principal component analysis:**

This function converts all the observations for each sample into variables called principal components. This simplifying 2 dimensional view reduce the dimensionality of the data set and exposing samples which are highly correlated with each other. High correlation between samples might imply that they share the same driving principle in defining the outcome of interest.

Press the show tools button if you like to edit the figure (Matlab version) .You can insert text box, color the clustered samples into the specific color (see lower figure) and more.

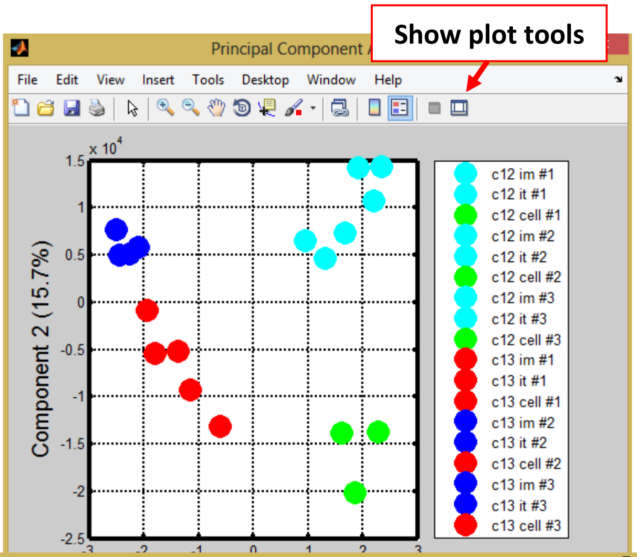

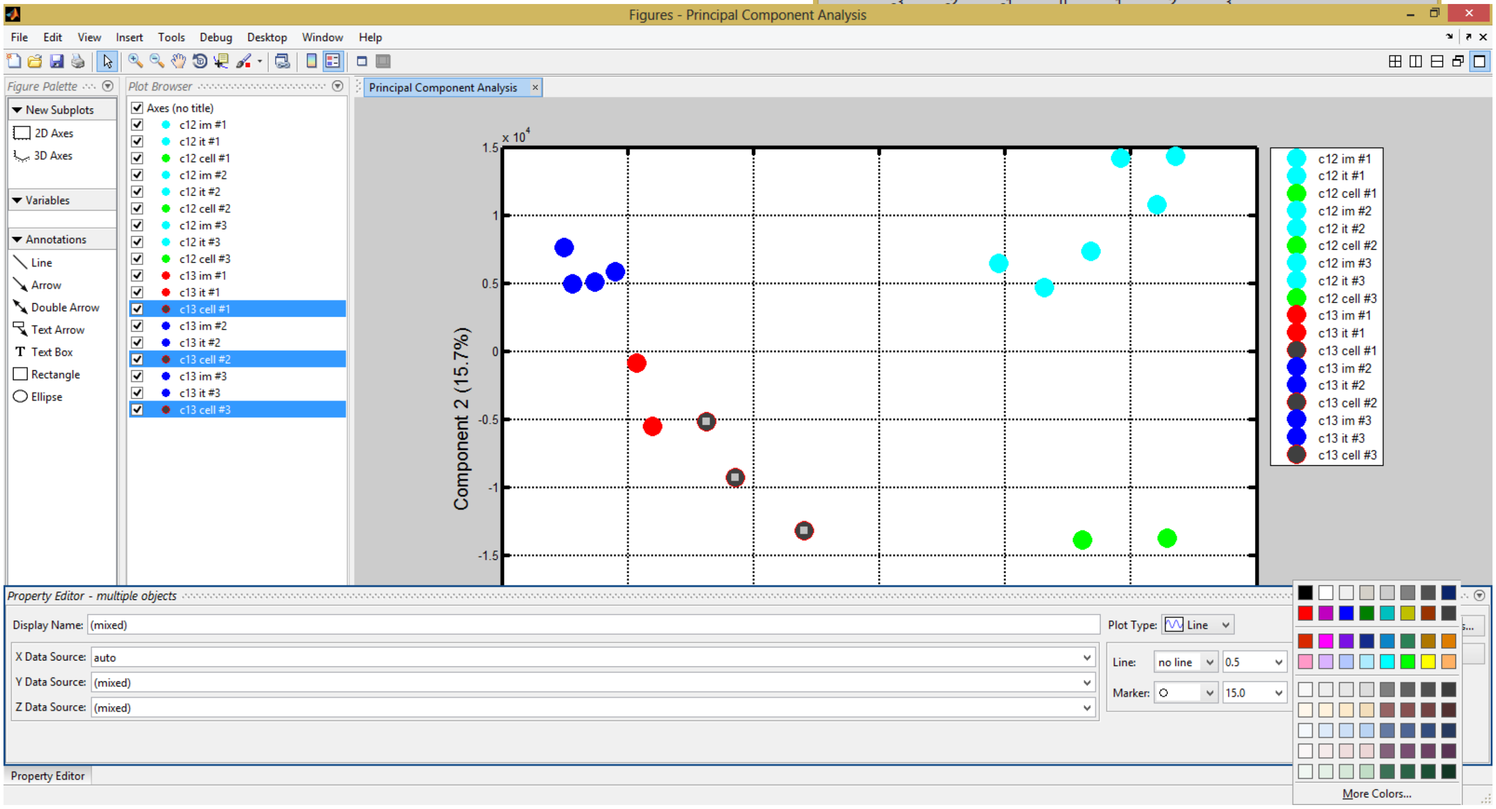

3.8 The probe interpretation and gene description panel

After finding the differentially expressed genes between the groups you will probably will want to know them better. After entering probe ID and execute the probe interpretation function you will see the gene symbol related to this probe, also if the bar plot or the single entry box plot are open, it will demonstrate automatically the information regarding your desired gene. (Probes IDs are case sensitive) .

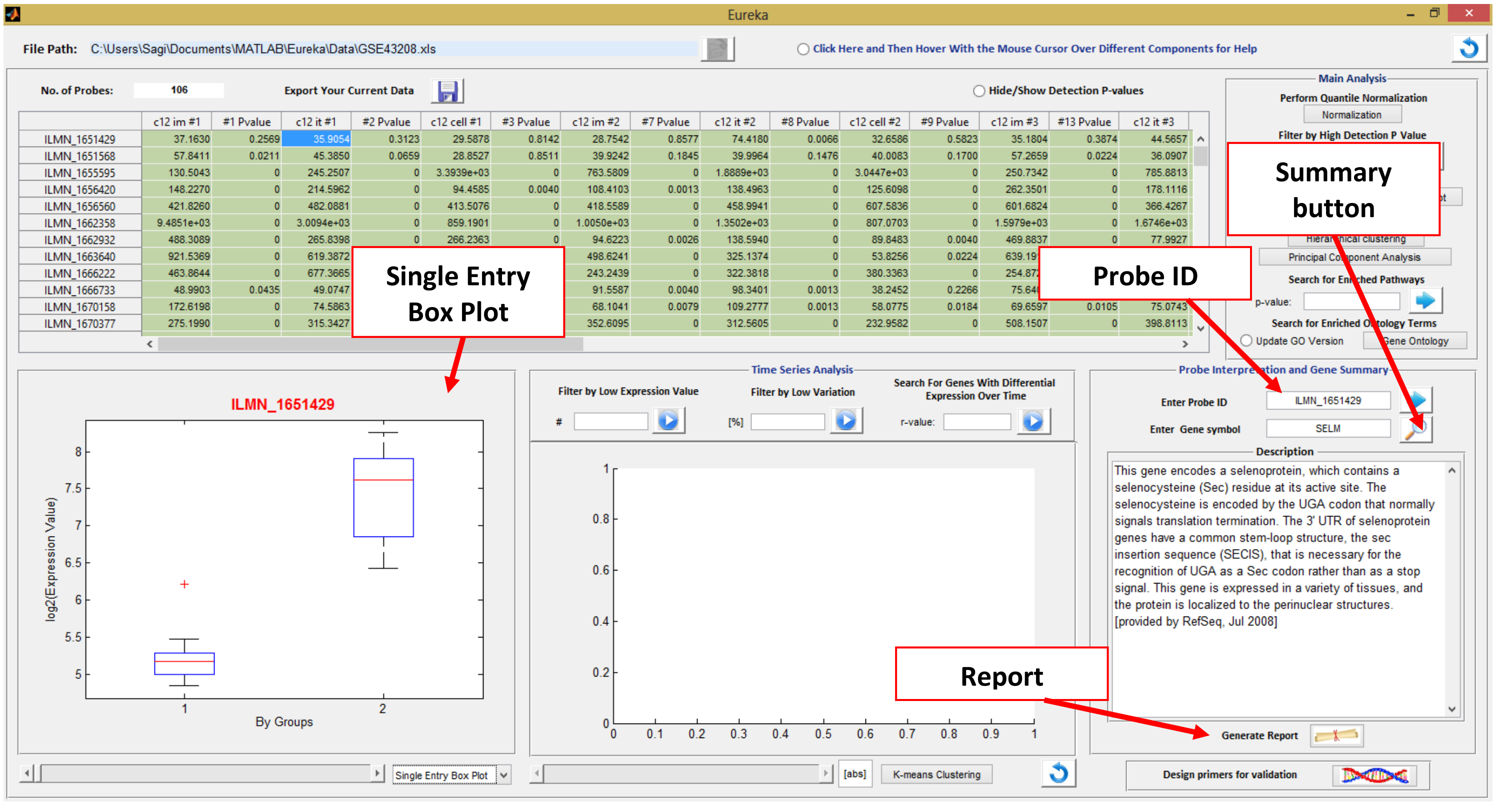

Please notice that you can press on the table on any cell within the row that indicates your probe of choice and then on the probe ID pushbutton to get its corresponding symbol (see blue marking on the picture)

Press the Summary button to withdraw the genes' description from NCBI (*http://www.ncbi.nlm.nih.gov/gene/*). You can also generate automated text report that will contain description about all the differentially expressed genes in your list. The description tool can be used as a standalone, no need to upload any data file. Just type your gene of interest and get its description. Although, if you wish to create a report about multiple genes please upload a file that was edited as in the picture.

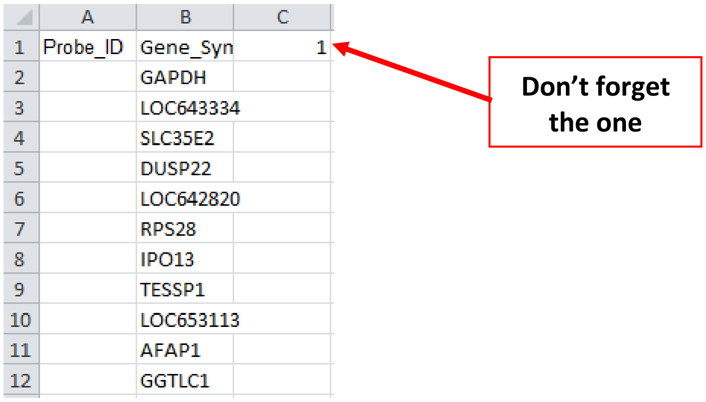

	A	B	C
1	Probe_ID	Gene_Syn	1
2		GAPDH	
3		LOC643334	
4		SLC35E2	
5		DUSP22	
6		LOC642820	
7		RPS28	
8		IPO13	
9		TESSP1	
10		LOC653113	
11		AFAP1	
12		GGTLC1	

3.9 Find the involvement of 'genes of interest' in specific pathways:

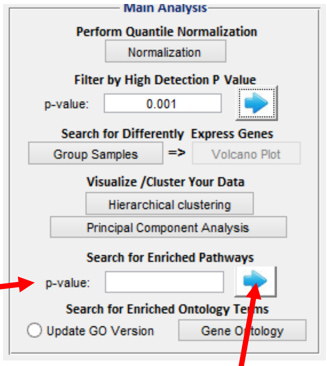

You can use Eureka-DMA to find if your differentially expressed genes participate in a common biologic or metabolic pathways which are published in the KEGG database (*www.genome.jp/kegg/pathway.html*) . For doing so please set the p-value threshold. The estimation for the statistic enrichment of genes in particular pathway is calculated according to the hypergeometric probability density test. (Please notice that Eureka-DMA's hypergeometric calculation is permissive since all known annotated genes with KEGG ID are taking into consideration as background list, also notice that the p-value is not adjusted for multiple comparisons)

After pressing on the Enriched pathways button, a new window will open, showing the statistically significant pathways in which your genes are involve.

Choose a pathway and press O.K to receive an illustration of the pathway you choose with your 'genes of interest' marked yellow.

You can change the color by simply changing the word yellow to blue, red etc.

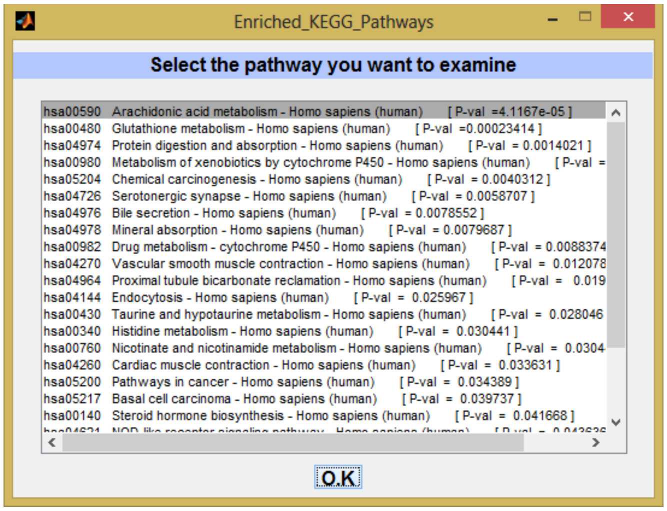

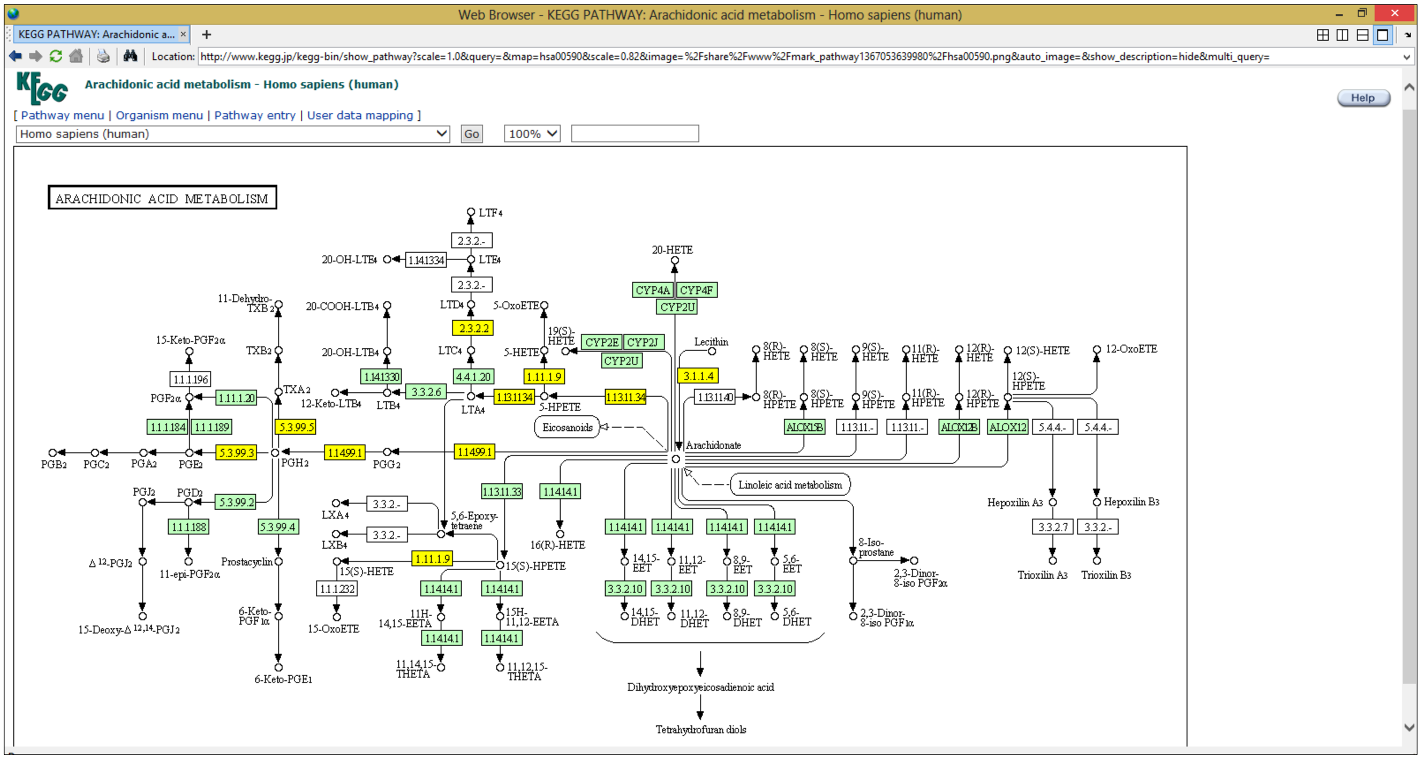

You can use the KEGG pathway function with any list of genes; just edit your data file as illustrated in the figure in the probe interpretation and gene summary panel section (previous page) before loading the file into Eureka-DMA.

3.10 Check for enrichment of gene ontology:

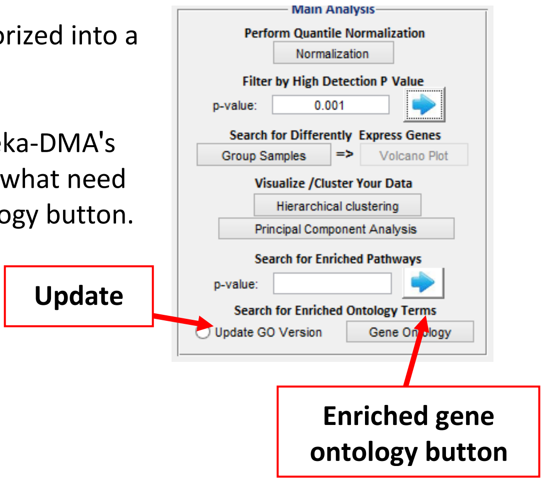

You can use Eureka-DMA to find if your differentially expressed genes can be categorized into a common gene ontology which are published in the gene ontology database (*www.geneontology.org/)*. Since the gene ontology database is updated frequently Eureka-DMA offers an option for updating the gene ontology association file in Eureka-DMA's Annotation folder. Once in a while press the update GO version radio button to see what need to be done. To execute the search for enriched gene ontology, press the gene ontology button. A new window will appear.

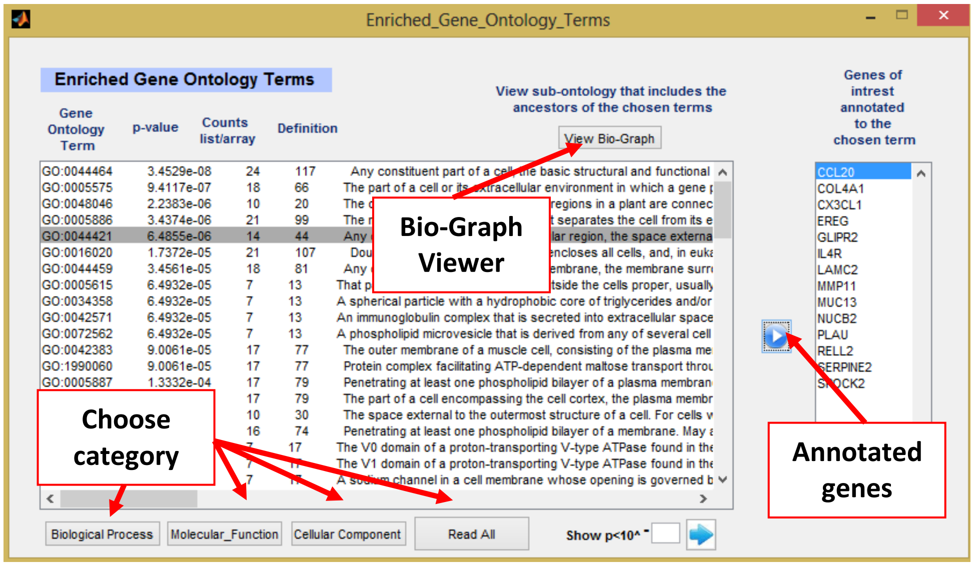

First choose the gene ontology category of which you want to describe the genes (In terms of their association in biological processes, cellular components and/or molecular functions). After doing so, the list will be populated with the appropriate significant enriched terms related to your 'genes of interest'. The statistic enrichment of gene ontology terms is calculated according to the hypergeometric probability density test. The background list in this calculation is the list of genes in your data before the search for differentially expressed genes. (After executing one or more of the following filters: low value filter, detection p-value filter or low variation filter).

You can now choose one or more terms in order to check which genes from your differentially expressed genes populate this term or to create a bio-graph and inspect the significant gene ontology terms by a sub-ontology that includes the ancestors of the terms (see figure below). In this bio-graph, intense red colors indicate low p-values while yellow indicate higher p-values. White color indicate that this term is not statistically enriched.

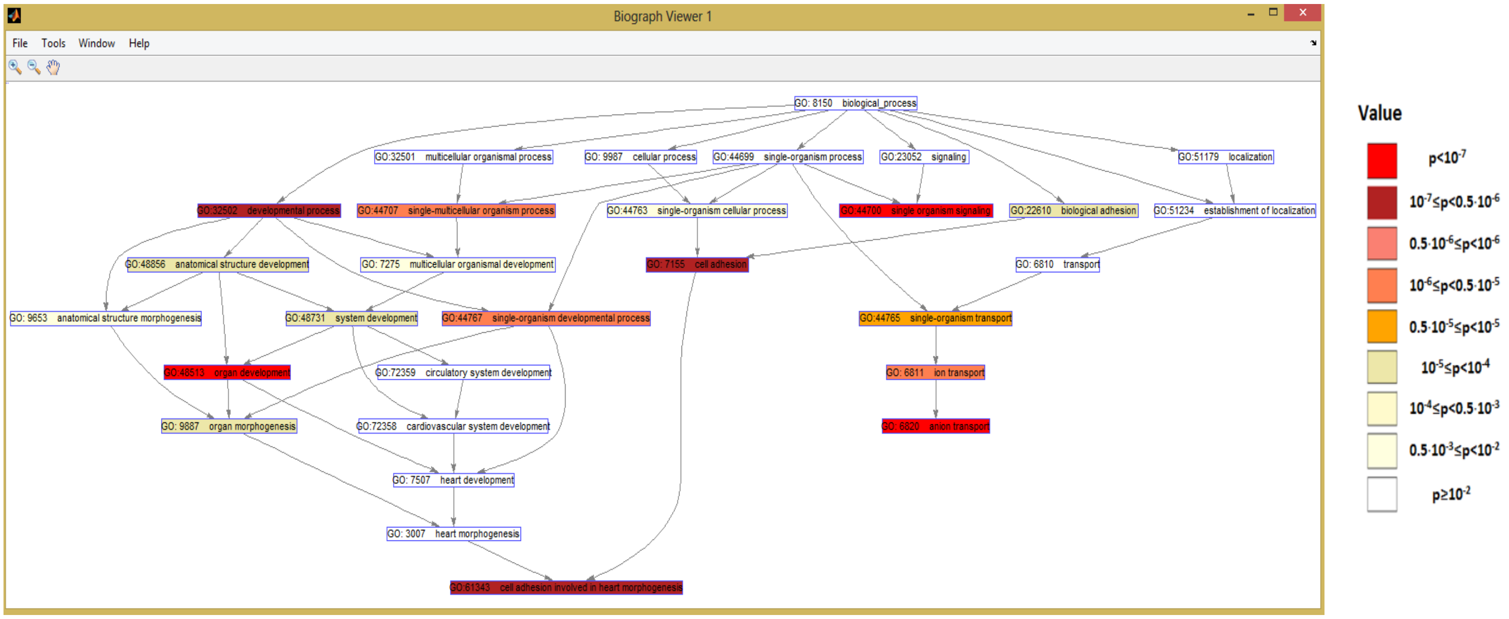

3.11 Work only on the up-regulated genes or the down regulated genes

Although its main purpose is different (see section 3.12) you can use the K-means clustering function in order to divide your list of differentially expressed genes into two. Write the number '2' in the appropriate box and execute the K-means clustering function in order to differentiate between those genes which their expression is elevated in group #1 and those genes which their expression is elevated in group #2. Use the K-means slider to navigate between the 2 sets of genes. You can also open the Box plot to get a better view on the gene set distributions. Press the k-means undo button to get back and work on all the differently expressed genes.

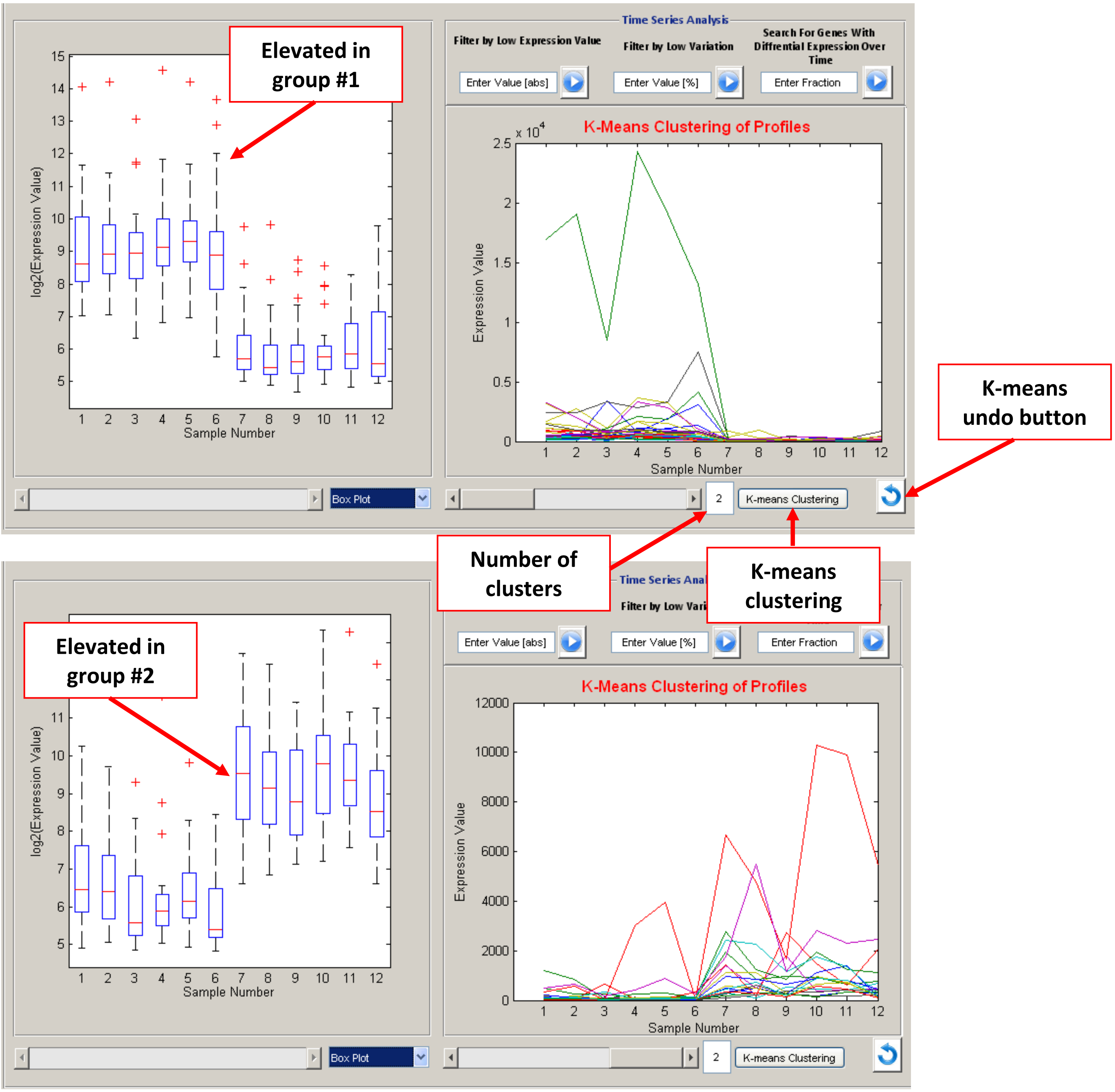

3.12 Time series analysis/ low variation filter

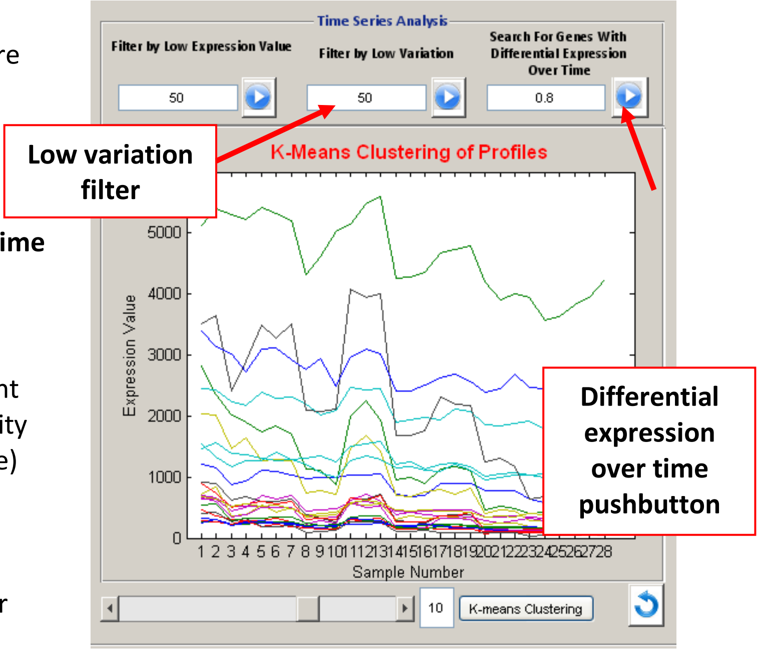

Time series data often contain more than two groups of samples, (Each group is a different time point) and therefore has to be analyzed differently.

very important: edit your data file in a way that the first samples indicates the first time point be on the left of your data table, and the samples accounting for the last time point are last, on the right.

Search for those probes/genes which have differential expression over time by using the pearson product-moment correlation coefficient (Pearson's r) to estimate the similarity between each probe/gene expression across samples (time) and the y=x equation.

0<Pearson's r<1 account for those probes/genes that their expression is elevated over time while

-1<Pearson's r<0 account for those probes/genes that their expression is reduced over time.

Setting a threshold of 0.8 for example will get you those probes/genes that have Pearson's r<-0.8 and those probes/genes that have Pearson's r>0.8.

After pushing the 'Differential expression over time push button' a new window will appear. In this window you need to inform how many time points are there in your data and how many replicated in each time point. In the example to the right there are 6 time points each is associated with 3 samples.

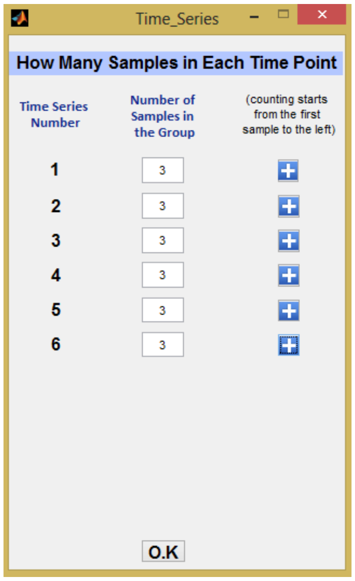

Press O.K to execute the function and find probes/genes which have differential expression over time.

It is recommended to start with low r-values and then use the Low variation filter to remove probes/genes that are changing "too little"

For example, set a percentile of 50 to remove from further analysis 50% of the entries which have the lower variation across the samples

- This function also suitable for analysis of different kinds of data sets in which more than 2 groups of samples are available (e.g. different drug concentration)

3.13 Design primers to validate your results.

Reverse transcription quantitative PCR is often used to validate gene expression measurements from DNA microarray experiments. For that reason, Eureka-DMA offers a primer design GUI.
You can either import the list of genes from the main GUI or used this tool as a standalone without the requirement of any previous analysis by just entering gene symbol. Gene symbols are case sensitive so if for example you want to get the sequence for the human isoform of actin beta, enter ACTB and for the mouse isoform enter Actb. If gene contains more than one transcript, you will see their different NCBI accession numbers in the appropriate list box. Choose one to reveal its sequence.

With multiple sliders and checkboxes you can control many of the desire primers parameters which are essential for successful reactions. Afterwards, the impact of individual filters can be observed in a 2 dimensional plot that can also reveal the existence of problematic regions within the cDNA transcript.

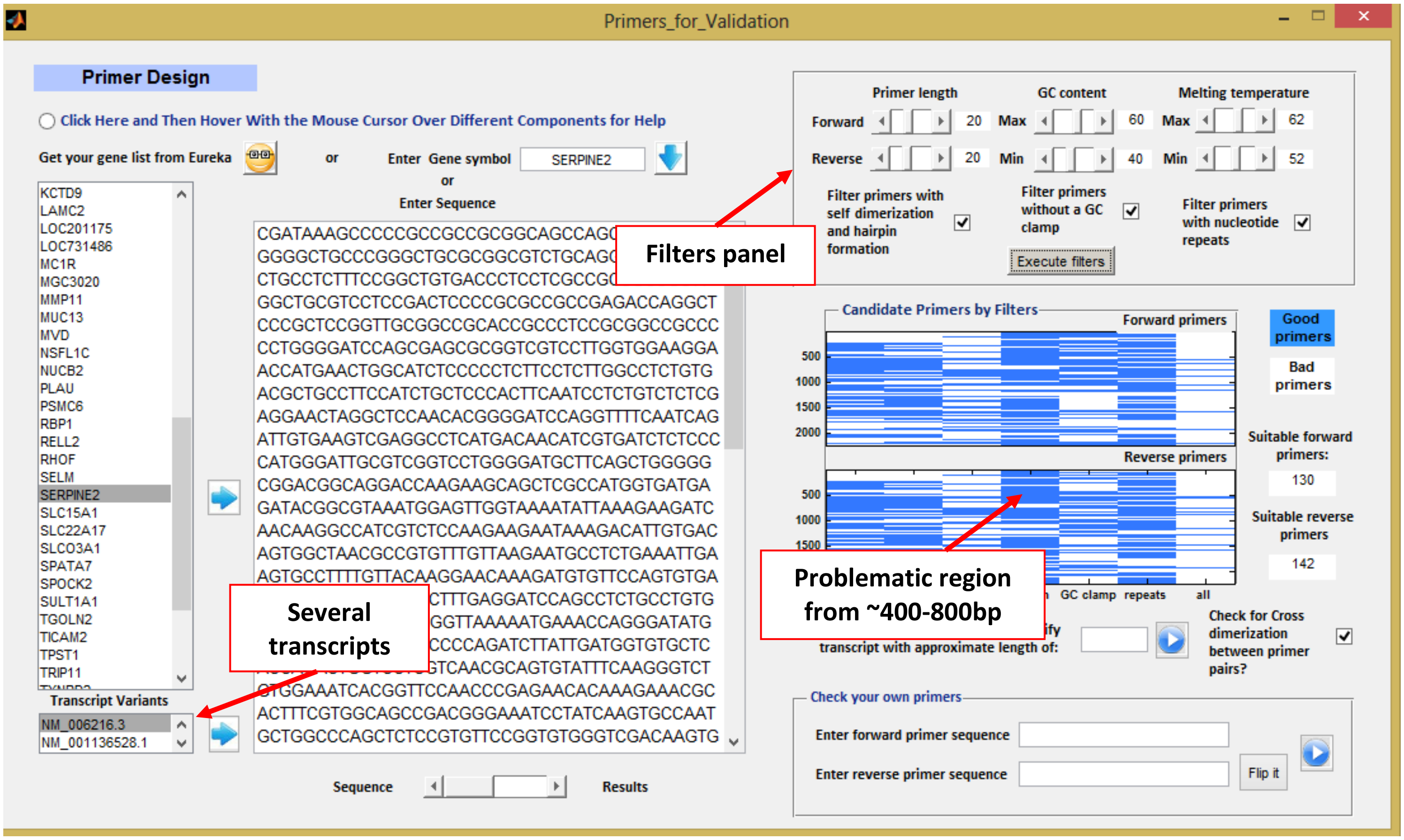

You can also design manually your own primers. If you chose to do so, don't forget to check the filters' sliders in the filters panel. Higher sliders values refers to your forward primer and the lower sliders values refers to the reversed primers. Also look at the check boxes. If they are checked, it means that both primers pass the appropriate filter

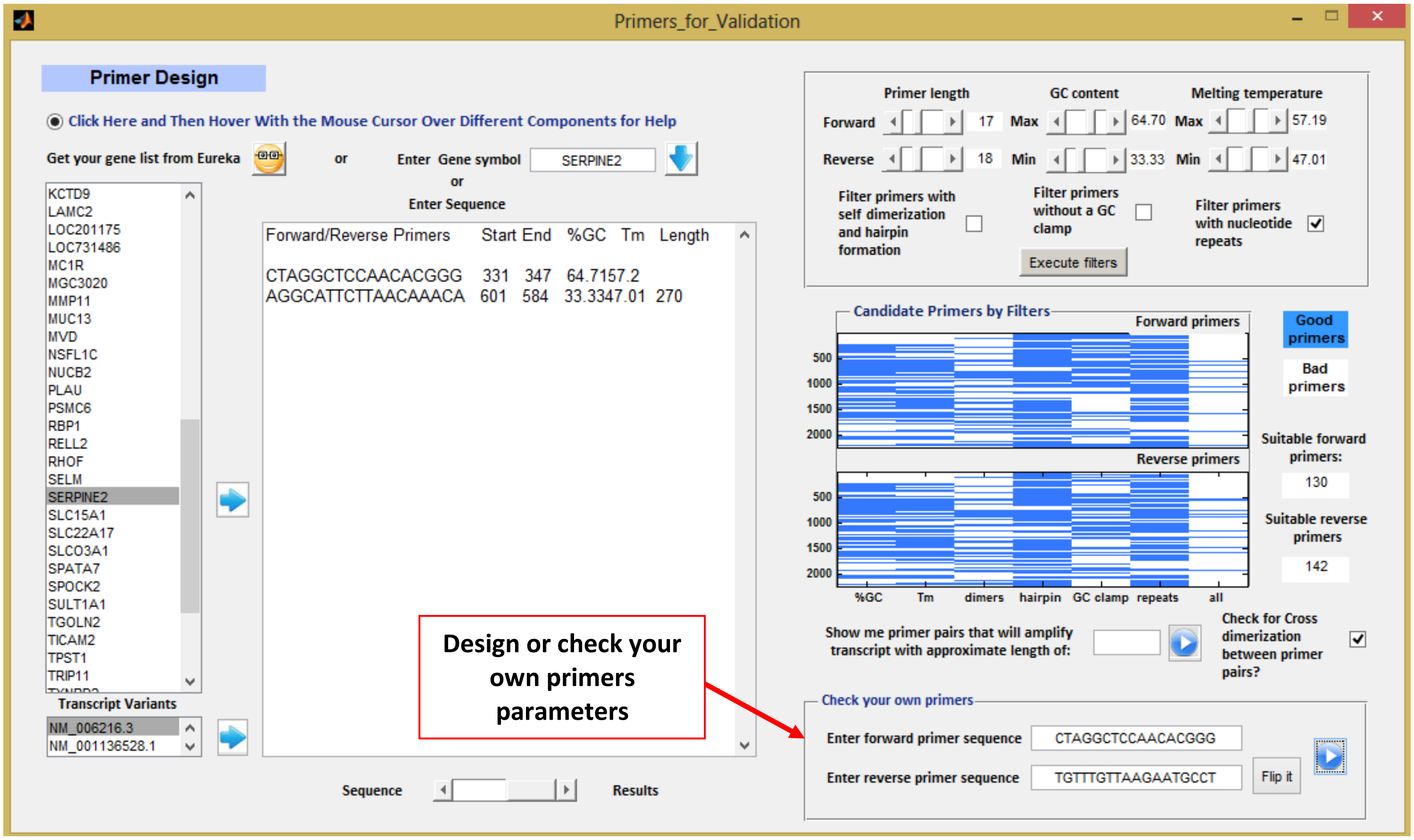

Thank you for reading the Tutorial
