# Supplementary figures and images for: Eureka-DMA: an easy-to-operate graphical user interface for fast comprehensive investigation and analysis of DNA microarray data
Source: BMC Bioinformatics. 2014 Feb 24;15:53. doi: 10.1186/1471-2105-15-53 (PMC3938137; doi:10.1186/1471-2105-15-53)

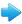

Supplement: Additional file 3 — ZIP archive file that contains all the software files needed in order to run Eureka-DMA in the MATLAB environment. This archive contains the code. [file 1471-2105-15-53-S3.zip › Eureka-DMA/Icons/Execute.png]

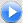

Supplement: Additional file 3 — ZIP archive file that contains all the software files needed in order to run Eureka-DMA in the MATLAB environment. This archive contains the code. [file 1471-2105-15-53-S3.zip › Eureka-DMA/Icons/Execute2.png]

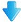

Supplement: Additional file 3 — ZIP archive file that contains all the software files needed in order to run Eureka-DMA in the MATLAB environment. This archive contains the code. [file 1471-2105-15-53-S3.zip › Eureka-DMA/Icons/ExecuteDown.png]

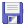

Supplement: Additional file 3 — ZIP archive file that contains all the software files needed in order to run Eureka-DMA in the MATLAB environment. This archive contains the code. [file 1471-2105-15-53-S3.zip › Eureka-DMA/Icons/ExportTable.png]

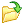

Supplement: Additional file 3 — ZIP archive file that contains all the software files needed in order to run Eureka-DMA in the MATLAB environment. This archive contains the code. [file 1471-2105-15-53-S3.zip › Eureka-DMA/Icons/LoadFile.png]

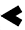

Supplement: Additional file 3 — ZIP archive file that contains all the software files needed in order to run Eureka-DMA in the MATLAB environment. This archive contains the code. [file 1471-2105-15-53-S3.zip › Eureka-DMA/Icons/move left.png]

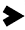

Supplement: Additional file 3 — ZIP archive file that contains all the software files needed in order to run Eureka-DMA in the MATLAB environment. This archive contains the code. [file 1471-2105-15-53-S3.zip › Eureka-DMA/Icons/move right.png]

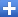

Supplement: Additional file 3 — ZIP archive file that contains all the software files needed in order to run Eureka-DMA in the MATLAB environment. This archive contains the code. [file 1471-2105-15-53-S3.zip › Eureka-DMA/Icons/Plus.png]

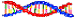

Supplement: Additional file 3 — ZIP archive file that contains all the software files needed in order to run Eureka-DMA in the MATLAB environment. This archive contains the code. [file 1471-2105-15-53-S3.zip › Eureka-DMA/Icons/Primers.png]

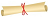

Supplement: Additional file 3 — ZIP archive file that contains all the software files needed in order to run Eureka-DMA in the MATLAB environment. This archive contains the code. [file 1471-2105-15-53-S3.zip › Eureka-DMA/Icons/Report.png]

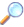

Supplement: Additional file 3 — ZIP archive file that contains all the software files needed in order to run Eureka-DMA in the MATLAB environment. This archive contains the code. [file 1471-2105-15-53-S3.zip › Eureka-DMA/Icons/Search.png]

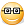

Supplement: Additional file 3 — ZIP archive file that contains all the software files needed in order to run Eureka-DMA in the MATLAB environment. This archive contains the code. [file 1471-2105-15-53-S3.zip › Eureka-DMA/Icons/smile.PNG]

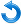

Supplement: Additional file 3 — ZIP archive file that contains all the software files needed in order to run Eureka-DMA in the MATLAB environment. This archive contains the code. [file 1471-2105-15-53-S3.zip › Eureka-DMA/Icons/Undo.png]
